# YAP9/A20 complex suppresses proinflammatory responses and provides novel anti-inflammatory therapeutic potentials

**DOI:** 10.3389/fimmu.2022.914381

**Published:** 2022-08-15

**Authors:** Fengyuan Mandy Yang, Liya Shen, Dengxia Denise Fan, Yaqin Bai, Bizhou Li, Jongdae Lee

**Affiliations:** State Key Laboratory of Respiratory Diseases, School of Basic Medical Sciences, Guangzhou Medical University, Guangzhou, China

**Keywords:** YAP9, A20, PRD, TAD, ZF7, TNF, anti-inflammatory peptides

## Abstract

Innate anti-inflammatory mechanisms are essential for immune homeostasis and can present opportunities to intervene inflammatory diseases. In this report, we found that YAP isoform 9 (YAP9) is an essential negative regulator of the potent inflammatory stimuli such as TNFα, IL-1β, and LPS. YAP9 constitutively interacts with another anti-inflammatory regulator A20 (TNFAIP3) to suppress inflammatory responses, but A20 and YAP can function only in the presence of the other. YAP9 uses a short stretch of amino acids in the proline-rich domain (PRD) and transactivation domain (TAD) suppress the inflammatory signaling while A20 mainly uses the zinc finger domain 7 (ZF7). Cell-penetrating synthetic PRD, TAD, and ZF7 peptides act as YAP9 and A20 mimetics respectively to suppress the proinflammatory responses at the cellular level and in mice. Our data uncover a novel anti-inflammatory axis and anti-inflammatory agents that can be developed to treat acute or chronic conditions where TNFα, IL-1β, or LPS plays a key role in initiating and/or perpetuating inflammation.

## Introduction

Acute and chronic inflammatory diseases present a significant personal and societal burden (1). Activation of immune responses by TNF, IL-1, and LPS is essential to initiate the defense against infections and/or injuries. However, despite the presence of many checks and balances to prevent hyper- or chronic inflammation (2, 3), acute hyper-inflammation and chronic inflammation throw the normal immune response into an uncontrollable and dangerous condition. The cellular responses to external stimuli are influenced by the mechanical inputs within the microenvironment such as shear stress, pressure, and tensile force (4). The Hippo signaling pathway is the major mechanosensor and the YAP1/TAZ complex as a co-transcription activator is the major downstream effector controlling various physiological events including cell proliferation and tissue regeneration (5).

The role of YAP1 on inflammatory responses is not clearly defined yet. It was reported to suppress inflammation in some setting (6, 7) while it enhances in other settings (8, 9). Mechanistically, YAP1, mainly as a transcription regulator, was reported to enhance the response to LPS/IFNγ through interaction with the histone deacetylase 3 (HDAC3)-nuclear receptor corepressor 1 (NCoR1) repressor complex (9), or suppress the TNF/IL-1 responses as a co-transcription repressor with HDAC7 (10) and the anti-viral response by blocking dimerization of the transcription factor IRF3 (7). YAP1 was also shown to suppress activation and anti-viral responses of T cells by impeding translocation of NFAT1 into the nucleus (11). How the same protein displays the mirror-opposite functions has yet to be addressed. Human YAP1 is expressed in up to 8 isoforms (12), but the precise role for each isoform is not well defined, especially in inflammation. There is a possibility that different isoforms play the opposite roles in the response to inflammatory stimuli.

We investigated how cytoskeletal tension influences the inflammatory responses at the cellular level by comparing the TNF-induced gene expression in 2D culture (high tension) and 3D culture (low tension). The magnitude of TNF-induced gene expression was significantly higher in 3D culture and YAP1 was responsible for the difference in the TNF response as a negative signaling regulator rather than a co-transcription activator. YAP1 isoform 9 (YAP9 herein) suppressed the cellular responses to TNF, IL-1, and LPS whereas isoform 2 (YAP2) enhanced the responses. Both the proline-rich domain (PRD) and the full-length TAD are required the suppressive function of YAP9 since PRD-mutation enhances inflammatory responses and YAP2 lacks a short segment in TAD. Mechanistically, both YAP2 and YAP9 proteins interact, but YAP9 with a higher affinity, with the anti-inflammatory regulator A20 (TNFAIP), which utilizes the zinc finger domain 7 (ZF7) for its suppressive function (13, 14). However, both YAP9 and A20 must be present together to attenuate the inflammatory responses since neither one can suppress the inflammatory response in the absence of another. Synthetic PRD, TAD, and ZF7 peptides alone or together suppress the inflammatory responses *in vitro* and *in vivo* by binding to the downstream signaling molecules such as TAK1, RIP, and NEMO. Our data present a new anti-inflammatory mechanism and a novel class of anti-inflammatory agents.

## Results

### Cell culture matrices regulate the TNFα response

**Figure 1:**
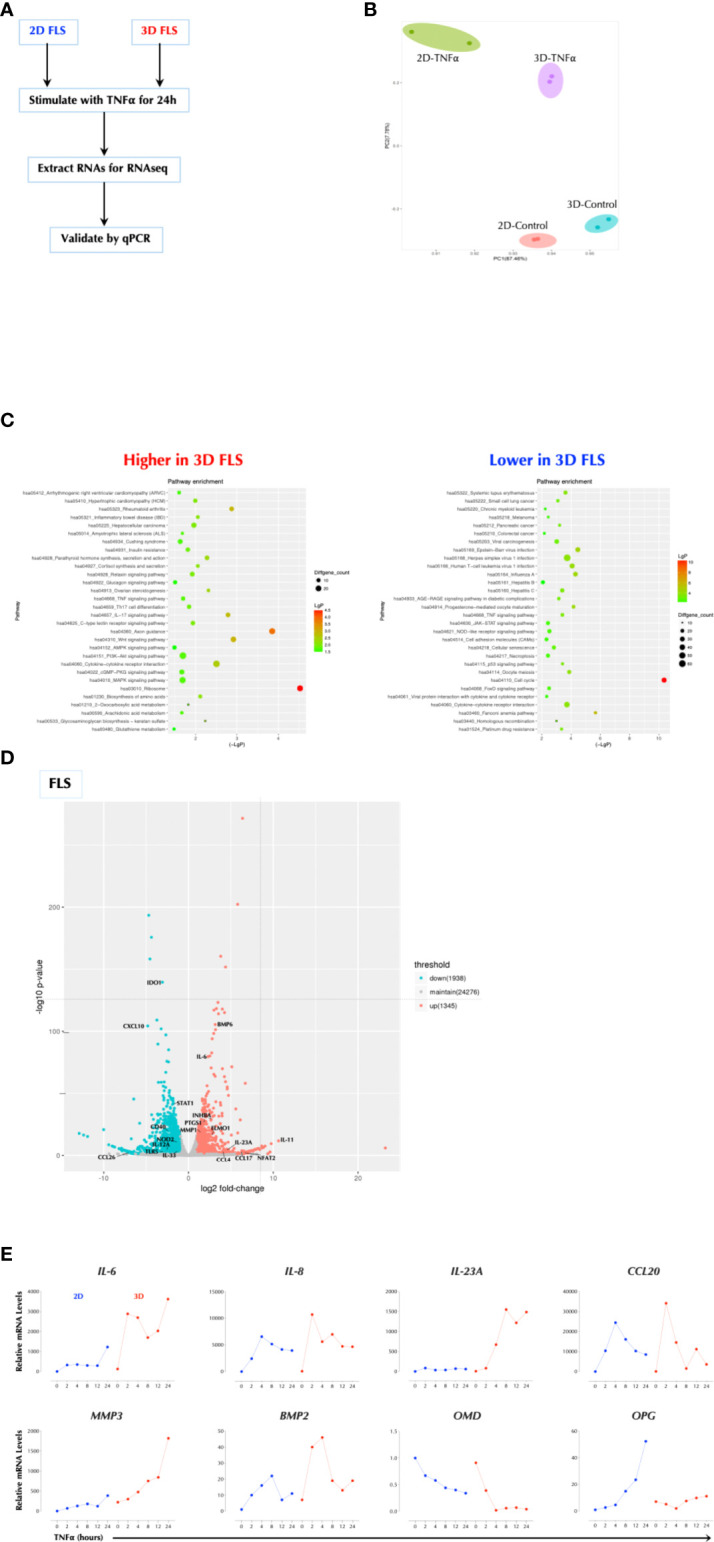
Cell Culture Matrix Regulates the TNF response. **(A)** Experimental schema. FLS were cultured in plastic tissue culture plate (2D) for 2 days or Vitrogel for 7 days and stimulated with TNFα (1ng/ml) for 2h in duplicates. Total RNAs were collected and subjected to RNAseq. **(B)** Principal component analysis (PCA) of RNAseq data. **(C)** Pathway analysis of RNAseq data. **(D)** A volcano plot of differential induced genes. **(E)** Validation of RNAseq data by qPCR analysis. FLS were cultured in plastic tissue culture plate (2D) for 2 days or Vitrogel for 7 days and stimulated with TNFα (1ng/ml) for the indicated time periods. The results are the representative of several similar experiments.

Since FLS (fibroblast-like synoviocytes) play an important role in the pathogenesis of rheumatoid arthritis (RA) (15), we investigated whether TNFα response in FLS is influenced by the matrix in which FLS were cultured. We measured the response to TNFα in FLS seeded either on a plastic culture plate (2D) or in a hydrogel matrix (Vitrogel™, 3D) by RNAseq (**Figure 1A**). The gene expression patterns were distinct in the two conditions and further differentiated after TNFα stimulation (**Figure 1B**). The genes involved in the inflammatory pathways were up-regulated in 3D over 2D culture after stimulation including RA, inflammatory bowel disease (IBD), and IL-17 signaling and differentiation, while the cell cycle-related genes were most notably down-regulated in 3D culture compared to 2D (**Figure 1C**). Among the most highly up-regulated genes in 3D over 2D included IL-6, IL-23A, and PTGS1 (**Figure 1D**). The qPCR analysis validated the sequencing results and showed that the induction level of several genes (e.g. IL-6, IL-23A, MMP3) by TNFα in 3D FLS was several magnitudes higher than that in 2D FLS while OPG was not induced at all by TNFα in 3D FLS (**Figure 1E**). The effects of 3D culture on the TNFα response in FLS was obvious within two days of culture (**Supplementary Figure 1A**). In addition, Matrigel™ culture had a similar effect as Vitrogel (**Supplementary Figure 1B**), indicating it is not specific to a particular matrix. The enhancement effect of 3D culture on the TNFα was not unique to FLS or a particular set of genes since the effect was reproduced in the human colonic carcinoma cell line HCT-8 with enhancement of all the measured genes (**Supplementary Figure 1C**). This was not due to an enhanced proliferation because all cell types divided much more slowly in 3D culture than 2D (data not shown) as suggested by the decreased level of cell-cycle-related genes. These data demonstrate that the cellular response to TNFα is influenced by the matrix in which they are cultured.

### Cytoskeletal tension suppresses the TNFα response *via* YAP1

**Figure 2:**
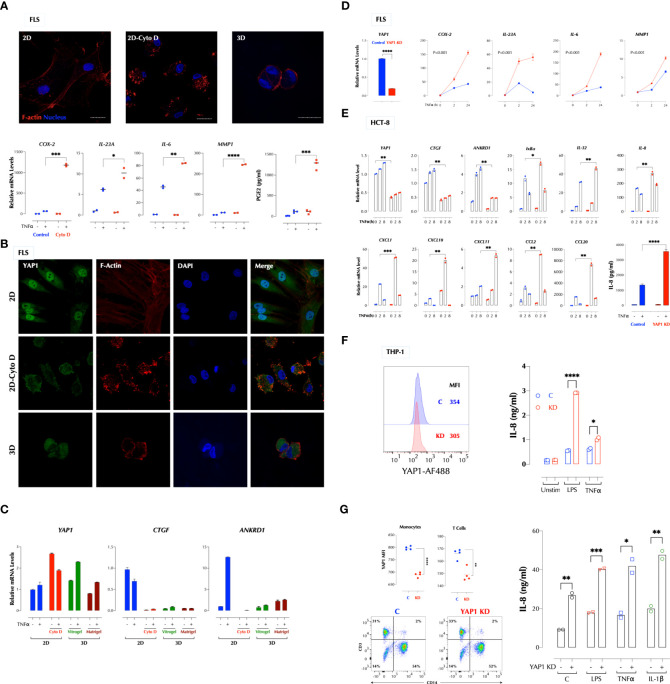
A high cytoskeletal tension suppresses the TNF response *via* YAP1. **(A)** Cytochalasin D (CytoD) disrupts actin polymerization and enhances the TNF response in FLS. FLS in 2D were treated with or without CytoD (1µg/ml) for 4h and stimulated with TNFα (1ng/ml) for 2h. PGE2 was measured by ELISA and the gene expression by qPCR. **(B)** Cytoskeletal tension regulates the YAP1 localization. FLS in 2D were treated with or without CytoD (1µg/ml) for 4h and FLS in 3D were cultured in Vitrogel for 7 days before staining YAP1 for confocal imaging. **(C)** A low cytoskeletal tension inhibits expression of YAP1 target genes in FLS. FLS in 2D were treated with or without CytoD (1µg/ml) for 4h and FLS in 3D were cultured in Vitrogel or Matrigel for 7 days before stimulation with TNFα (1ng/ml) for 2h for qPCR. PGE2 was measured after 24h stimulation by ELISA. **(D-G).** YAP1 knockdown in 2D enhances the TNF response in FLS **(D)**, HCT-8 **(E)**, THP-1 **(F)**, and human PBMCs **(G)**. YAP1 was silenced by siRNA and cells were stimulated with TNFα (1ng/ml) as indicated. The gene expression levels were measured by qPCR, IL-8 protein levels by ELISA, and YAP1 protein levels by flow cytometry. * P < 0.05, ** P < 0.01, *** P < 0.001, ****: 0.0001.

We next investigated whether stiffness of a matrix plays a role in the TNFα response since the hydrogel is significantly softer than the plastic surface. Stress actin fibers are stretched long in 2D FLS but short in 3D FLS. Cytochalasin D (CytoD) is a cell-permeable inhibitor of actin polymerization and binds to Fiber-actin (F-actin) polymer and prevent polymerization of actin monomers. CytoD treatment of 2D FLS disrupted actin polymerization and significantly enhanced the TNFα response (**Figure 2A**), similar to 3D culture. TNFα did not affect actin fibers even after overnight stimulation (data not shown). Since the transcriptional co-activator YAP1 (Yes-associated protein 1) mediates many mechanical cues including extracellular matrix stiffness (4), we next investigated whether YAP1 regulates the TNFα response. YAP1 in 2D FLS was mostly localized in the nucleus but mainly in the cytosol of 3D FLS, and YAP1 in 2D FLS translocated to the cytosol by the CytoD treatment (**Figure 2B**), indicating that YAP1 localization is regulated by the cytoskeletal tension supported by actin fibers. The expression of the YAP1 target genes CTGF and ANKRD1 was consistent with the YAP1 localization patterns (**Figure 2C**). Knockdown (KD) of YAP1 in 2D FLS significantly enhanced the TNFα response (**Figure 2D**) without affecting F-actin formation (**Supplementary Figure 2A**), indicating a suppressive role of YAP1 in the TNFα response not through actin formation. The suppressive function of YAP1 was not restricted to FLS or TNFα. YAP1 depletion enhanced the TNFα response in the epithelial cell lines HCT-8 (**Figure 2E**) and 293-T (data not shown). We next tested the role of YAP1 in suspension cells that cytoskeletal tension is relatively weak compared to adherent cells. Surprisingly, the TNFα and LPS response in the human monocytic cell line THP-1 (**Figure 2F**) and the IL-1β, TNFα and LPS response in the human PBMCs (**Figure 2G**) were also enhanced by YAP1 KD. This indicated that YAP1 suppresses inflammatory responses with or without mechanical cues. These data together established YAP1 as a suppressor of multiple pro-inflammatory stimuli. Herein, YAP1 or YAP refers to all isoforms whereas YAP2 and YAP9 are YAP isoforms 2 and 9 respectively.

### Suppression of the TNFα signaling by YAP1 is not dependent on its transcriptional activity

**Figure 3:**
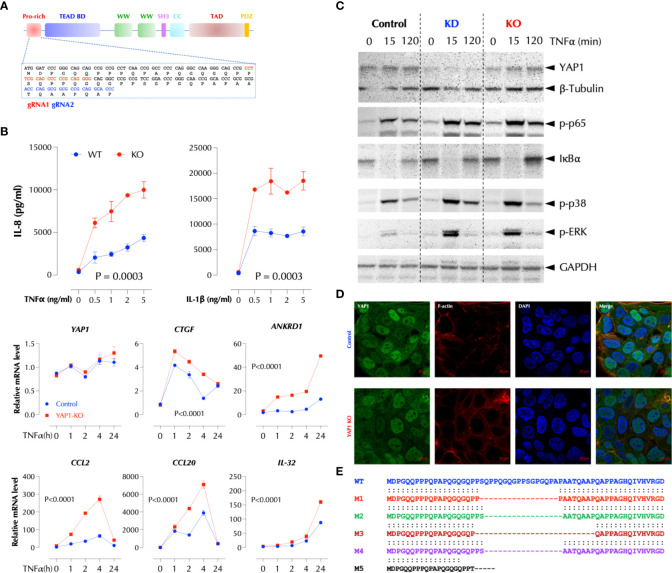
PRD is essential for the suppressive function of YAP1. **(A)** Crispr/Cas9-mediated KO strategy of YAP1. **(B)** YAP1 KO enhances TNF- and IL-1-induced IL-8 expression and TNF-induced genes in HCT-8. YAP1 KO cells were stimulated with the indicated amount of TNFα or IL-1β overnight and IL-8 levels were measured by ELISA. TNF-induced gene expression was measured by qPCR after stimulation as indicated. The results are the representatives of two similar experiments. **(C)** YAP1 KD or KO enhances TNFα-induced activation of NF-κB and MAP kinases. Control, YAP1 KD or KO HCT-8 cells were stimulated with TNFα (2ng/ml) for the indicated time period and western blotting was performed with the indicated antibodies. **(D)** YAP1 in KO cells is localized normally and mostly in the nucleus. **(E)** Most YAP1 mRNAs in KO cells are edited with in-frame deletions. YAP1 mRNAs in KO cells were PCR-amplified, TA-cloned, and sequenced.

**Figure 4:**
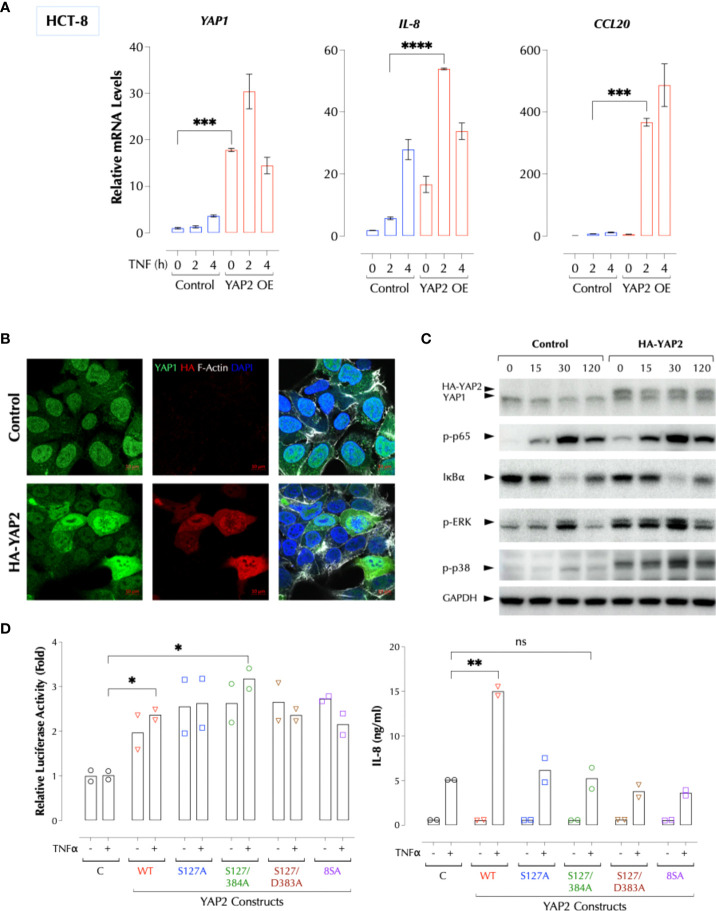
YAP2 enhances the inflammatory responses *via* a non-transcriptional activity. **(A)** YAP2 enhances the TNF response. The C or YAP2-OE HCT-8 cells were stimulated with TNFα (2ng/ml) for 2-4h and the mRNA levels of the indicated genes were measured by qPCR. **(B)** YAP2 is localized mostly in the nucleus in 2D HCT-8 cells similarly to the endogenous YAP1. **(C)** YAP2 enhances the TNF signal transduction. Control DNA or HA-YAP2 transfected HCT-8 cells were stimulated with TNFα (2ng/ml) for the indicated time periods and activation of NF-κB and MAP kinases was measured by western blotting. **(D)** Non-transcription activity of YAP2 enhances the TNF response. The indicated YAP2 constructs were transfected with a YAP reporter and the YAP1-mediated transcriptional activity was measured with Dual-Luciferase^®^ Reporter Assay System and IL-8 by ELISA. * P < 0.05, ** P < 0.01, *** P < 0.001, ****: 0.0001, ns, not significant.

To further investigate how YAP1 suppresses the TNFα response, we performed Crispr/Cas9-mediated knockout of YAP1 using two gRNAs targeting the proline-rich domain (PRD) (**Figure 3A**). YAP1 KO in HCT-8 cells also significantly enhanced TNFα- or IL-1β-induced IL-8 production and TNFα-induced cytokines and chemokines at the transcription level (**Figure 3B**). Consistently activation of the NF-κB and MAP kinases upon TNFα stimulation was enhanced by either KD or KO of YAP1 (**Figure 3C**), indicating that YAP1 suppresses the proximal TNFα signaling. Unexpectedly, however, YAP1 KO cells still expressed the YAP1 mRNA and protein (**Figures 3B, C**) and the expression of YAP1 target genes (CTGF and ANKRD1) in the KO cell line was higher than that in control (**Figure 3B**), indicating YAP1’s co-transactivator function is enhanced. Mutant YAP1 localization in the KO cells was not different from that in WT cells (**Figure 3D**). To verify the gene editing, we sequenced 7 YAP1 mRNA clones from the KO cells. The results showed that all except for one (M5) had a short in-frame deletion of various sizes in PRD targeted by the two gRNAs, explaining the expression of YAP1 mRNA and protein in the KO cells (**Figure 3E**). Therefore, the cell line is a mixture of true YAP1 KO and PRD-deficient YAP1 (YAP1^ΔPRD^) cells. The data suggested that YAP1 suppresses the pro-inflammatory stimuli using PRD rather than its transcriptional activity as suggested (6) because the transactivation domain (TAD) of the YAP1 in-frame deletion mutants in KO cells is intact and the YAP1 target genes (CTGF, ANKRD1) are still expressed. Next, to test if ectopic YAP1 expression suppresses the TNFα response, we over-expressed a recombinant HA-YAP2 (16) (isoform 2). Contrary to the expectation and just like YAP1 depletion or PRD mutation, YAP2 over-expression (OE) enhanced the TNFα response (**Figure 4A**). HA-YAP2 cellular distribution was similar to that of the endogenous YAP1, mostly in the nucleus (**Figure 4B**) and it enhanced the TNFα signaling pathways (**Figure 4C**), indicating its global effect on the signaling pathways. However, mutations in the phosphorylation sites of YAP2 that increase its co-transcriptional activity (16) failed to enhance the TNF response (**Figure 4D**), indicating YAP2 enhances the TNF response *via* a non-transcriptional activity. These data together demonstrate that while YAP1 overall suppresses the inflammatory responses, a certain isoform (e.g. YAP2) enhances it, in both cases *via* a non-transcriptional activity.

### YAP1 utilizes two distinct domains to suppress inflammatory signals

**Figure 5:**
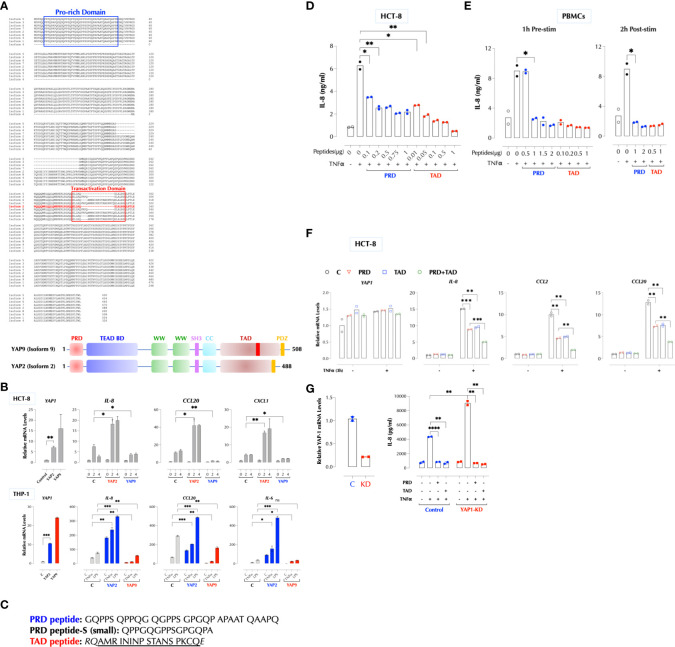
YAP1 suppresses the inflammatory responses using PRD and TAD. **(A)** Protein sequence alignment of human YAP1 isoforms. A difference between YAP2 and YAP9 lies within TAD. **(B)** YAP2 enhances but YAP9 suppresses the inflammatory responses. HCT-8 or THP-1 cells (2h stimulation) were transfected with HA-YAP2 or HA-YAP9 and stimulated as indicated and the gene induction levels were measured by qPCR. The results are representatives of 2 or more similar experiments. **(C)** The sequences of synthetic PRD, PRD-S, and TAD peptides. **(D)** PRD and TAD peptides inhibit TNF-induced IL-8 expression in a dose-dependent manner. HCT-8 cells were stimulated with TNFα (2ng/ml) with or without the indicated amount of the peptides overnight and IL-8 was measured by ELISA. The results are representatives of 2 or more similar experiments. **(E)** Human PBMCs were treated with the peptides 1h before or 2h after stimulation with TNFα (1ng/ml) overnight and IL-8 was measured by ELISA. The results are representatives of 2 similar experiments. **(F)** HCT-8 cells were stimulated with TNFα (2ng/ml) as indicated and IL-8 was measured by ELISA. **(G)** PRD and TAD peptide inhibit the TNF response in YAP1-deficient cells. Control or YAP1-KD HCT-8 cells were stimulated with TNFα (2ng/ml) as indicated, and IL-8 was measured by ELISA the next day. * P < 0.05, ** P < 0.01, *** P < 0.001, ****: 0.0001, ns, not significant.

Human cells express at least 8 different YAP1 isoforms (12). There are two major regions where YAP1 isoforms differ in the protein sequence, and PRD is conserved except for isoform 4. YAP2 lacks a small stretch of amino acids in the transactivation domain (TAD) along with isoforms 3, 5, and 8 (**Figure 5A**). Based on our data and the sequence homology, we hypothesized that the peptides in PRD and TAD mediate the suppressive activity of YAP1 and that PRD alone is insufficient. YAP9 having both PRD and the full-length TAD is then expected to suppress the TNFα response. Indeed, YAP9 suppressed TNFα-induced gene expression while YAP2 enhanced in THP-1 and HCT-8 cells (**Figure 5B**). The data confirmed that both PRD and the full-length TAD are required for YAP9 to suppress the inflammatory responses. If YAP9 inhibits but YAP2 promotes the TNF response as non-transcriptional regulators as our data indicate, we wondered why the TNF response is much higher when YAP proteins are mostly in the cytosol in 3D culture or CytoD-treated 2D culture. As mechanical stresses are known to influence the transcription patterns of different isoforms (17), we tested whether different YAP isoforms are expressed in 2D and 3D. Indeed, the predominant YAP isoforms (2, 3, 5, 8) in 3D or CytoD-treated 2D FLS were lacking the inhibitory TAD region although the total YAP mRNA levels were higher than 2D FLS, indicating the stoichiometry of YAP1 isoforms determines the inflammatory response (**Supplementary Figures 3A–D**).

The role of YAP1 in mouse cells on inflammatory responses is not clear. In one report, ectopic expression of YAP2 or YAP4 suppressed anti-viral immunity while YAP1 depletion enhanced it, which is consistent with our data (7). But in another report, depletion of YAP1 inhibited inflammatory responses in macrophages (8). When aligned with human YAP9, all mouse isoforms lack a stretch of 15 amino acids in PRD, similar to YAP1^ΔPRD^ in this report (**Figure 3E**), while two groups with or without the full TAD are similar to human YAP isoforms (**Supplementary Figure 3E**). When YAP1 was depleted in the mouse macrophage cell line RAW264.7, the LPS-induced expression of TNFα was lower than the control while the baseline TNFα expression was slightly higher than the control (**Supplementary Figure 3F**). Overall, it appears that mouse YAP isoforms are generally pro-inflammatory due to the lack of complete PRD.

Next, we tested the idea that synthetic PRD and TAD peptides can mimic the suppressive function of YAP9 (**Figure 5C**). Transfection of either peptide indeed the TNFα response in HCT-8 cells and TAD peptide was more potent than PRD (**Supplementary Figure 4A**). They also inhibited the response of human PBMCs to TNFα, IL-1β, or LPS (**Supplementary Figure 4B**). We also found that a shorter PRD peptide (PRD-Small) from one of mRNA clones in the KO cells (**Figure 3E**) also suppressed the TNF response (**Supplementary Figure 4C**). Because these peptides are highly hydrophobic with the hydrophobicity score of 17.92 for PRD and 21.16 for TAD peptide, we tested whether they can penetrate cells without an aid (18). In order to trace the cellular distribution, we generated biotinylated versions and found them still bioactive (**Supplementary Figure 4D**). Biotinylated PRD and TAD readily penetrated THP-1 cells and mostly detected near the cytoplasmic membrane and cytosol by confocal microscopy (**Supplementary Figure 4E**). Without transfection, both peptides inhibited TNFα-induced (**Figure 5D**), as well as IL-1β-induced IL-8 production in a dose-dependent manner in HCT-8 and THP-1 cells (**Supplementary Figures 4F, G**) as well as in human PBMCs (**Figure 5E**). The peptides were effective whether they were delivered to PBMCs before or 2 hours after stimulation (**Figure 5E**). Combination of two peptides inhibited better than each one alone (**Figure 5F**). In addition, PRD or TAD inhibited the TNF response in YAP1-deficient cells (**Figure 5G**), indicating they can replace YAP9 protein as hypothesized.

### YAP9 suppresses pro-inflammatory stimuli in collaboration with A20

**Figure 6:**
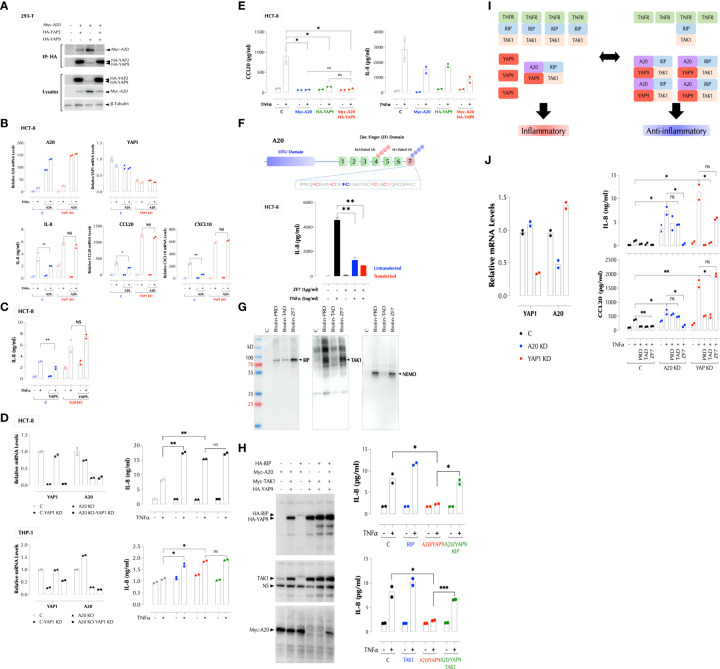
YAP1 and A20 collaboratively suppress the inflammatory responses. **(A)** YAP proteins constitutively interact with A20 and YAP9 has a higher affinity to A20 than YAP2. 293-T cells were transfected with the indicated expression constructs and immunoprecipitation (IP) and western blotting were performed as indicated. **(B)** YAP9 is required for A20 to suppress the TNF response. C and YAP1-KD HCT-8 cells with or without A20-OE were stimulated with TNFα (2ng/ml) for 2h or overnight for qPCR or IL-8 ELISA, respectively. **(C)** YAP9 requires A20 to suppress the TNF response. C and A20-KO HCT-8 cells were stimulated with TNFα (2ng/ml) overnight for IL-8 ELISA. **(D)** YAP1 or A20 single deficiency enhances the TNF response as much as YAP1/A20 double deficiency. YAP1 KD and/or A20 KO were performed in HCT-8 or THP-1 cells, and qPCR and ELISA were done after stimulation with TNFα overnight. **(E)** Over-expression of YAP9 or A20 alone suppresses the TNF response as much as YAP1/A20 together. HCT-8 cells were transfected as indicated and stimulated with TNFα (2ng/ml) overnight, followed by IL-8 and CCL20 ELISA. **(F)** ZF7 peptide in A20 suppresses the TNF response. The indicated ZF7 peptide was biotinylated and added to HCT-8 cells with or without peptide transfection. TNF-induced IL-8 expression was measured by ELISA. **(G)** PRD, TAD, and ZF7 peptides bind to the TNF signaling components, RIP, NEMO, and TAK1. Biotinylated peptides were used to pulldown cellular targets from HCT-8 total cell lysates. The results are a representative of 3 experiments. **(H)** RIP or TAK1 OE overcomes the A20/YAP9-mediated suppression of the TNF response. HCT-8 cells were transfected and stimulated with TNFα (2ng/ml) overnight, followed by IL-8 ELISA. **(I)** A stoichiometry model of YAP9/A20-mediated suppression of the TNF response. As A20 is induced by inflammatory stimuli, the number of A20-YAP9 complex increases, shifting the balance from inflammatory to anti-inflammatory response. **(J)** PRD and TAD require A20 to suppress the TNF response while YAP1 must be present for ZF7 to do the same. HCT-8 cells were transfected with the indicated siRNA and stimulated with TNFα (2ng/ml) overnight with the indicated peptides for IL-8 and CCL20 ELISA. * P < 0.05, ** P < 0.01, *** P < 0.001, ns, not significant.

We next investigated the mechanism by which YAP9 inhibits the signaling pathways activated by pro-inflammatory stimuli. Our data suggested that it involves a negative regulator(s) shared by IL-1β, TNFα and LPS. One such molecule is A20 also known as TNFAIP3 (TNFα-induced protein 3), which uses its zinc finger motif 7 (ZF7) to restrict the signaling by these stimuli (13, 14). We hypothesized YAP9 must be either parallel or downstream to A20 since its deficiency generates a pro-inflammatory response in the presence of A20. First, we tested by immunoprecipitation whether A20 and YAP physically interact. In 293-T cells, YAP2 was expressed consistently at a much higher level than YAP9 when the same amount of expression vector was transfected, and YAP9 elevated the A20 expression level compared to YAP2 when co-transfected (**Figure 6A**, lysates). Both YAP2 and YAP9 pulled down A20. However, YAP9 had a stronger affinity to A20 than YAP2 since a significantly less amount of YAP9 pulled down more A20 than YAP2 did (**Figure 6A**). In addition, when YAP2 and YAP9 are co-expressed with A20, HA pulled down mostly YAP2 and much less A20 than YAP2 or YAP9 alone, indicating that most of A20 is associated with YAP9 when all 3 are present (**Figure 6A**). These data suggest that the YAP9-A20 complex is the predominant form in the cells, thus ensuring an anti-inflammatory environment. Next, we investigated whether YAP9 or A20 can suppress the TNF response collaboratively. A20 was no longer able to suppress the TNF response in the absence of YAP (**Figure 6B**) and similarly YAP9 did not suppress the TNF response in the absence of A20 (**Figure 6C**). Furthermore, depletion of both YAP1 and A20 did not increase the TNF response above depletion of YAP1 or A20 alone in two different cell types (**Figure 6D**). Consistently, expression of A20 or YAP9 alone suppressed the TNF signal transduction as much as YAP9 and A20 together did (**Figure 6E**). As A20 uses ZF7 motif to suppress the inflammatory response, we tested whether ZF7 peptide acts as an inhibitor of inflammatory stimuli. Indeed, synthetic ZF7 (or biotinylated ZF7) inhibited TNF-induced IL-8 expression with or without being transfected (**Figure 6F**). ZF7 also penetrated cells without transfection (**Supplementary Figure 4E**). We found PRD, TAD and ZF7 peptides could be further shortened (**Supplementary Figure 4H**) and all 3 peptides inhibited the LPS response in the mouse macrophage cell line RAW264.7 (**Supplementary Figure 4I**).

As the YAP9-A20 complex inhibits different pro-inflammatory stimuli, we hypothesized that it interferes with the proximal signaling molecules common to TNFR1, TLR, and IL-1R. To identify molecules that they interact with, we performed a pulldown assay with biotinylated PRD, TAD, or ZF7 peptide as a bait. Biotinylated PRD, TAD, or ZF7 pulled down RIP (RIP1K), TAK1, and NEMO with a different affinity (**Figure 6G**). Since TNFR1, RIP, and TAK1 formed a signaling complex in the cells (**Supplementary Figure 5**), we tested whether ectopic expression of RIP or TAK1 can overcome the suppression imposed by the YAP9-A20 complex. Indeed, either RIP, or TAK1 expression relieved the suppression by the YAP9/A20 complex (**Figure 6H**), suggesting that it is a stoichiometry between the positive and negative signaling complexes that determines the signaling output (**Figure 6I**). Since the A20 expression level is relatively low but it is induced by the inflammatory stimuli such TNFα, IL-1β, and LPS, A20 can tip the balance from pro- and anti-inflammatory states before and after stimulation, respectively (**Figure 6I**). We did not find a significant change in the YAP1 expression after TNFα stimulation.

Just as A20 could not inhibit the TNF response in the absence of YAP1 (**Figure 6B**), ZF7 peptide was ineffective in YAP1-deficient cells (**Figure 6J**); Similarly, as YAP9 was unable to suppress the TNF response in the absence of A20 (**Figure 6C**), PRD and TAD peptides less effective in A20-deficient cells (**Figure 6J**).

### PRD, TAD, and ZF7 peptides are a novel class of anti-inflammatory agents

**Figure 7:**
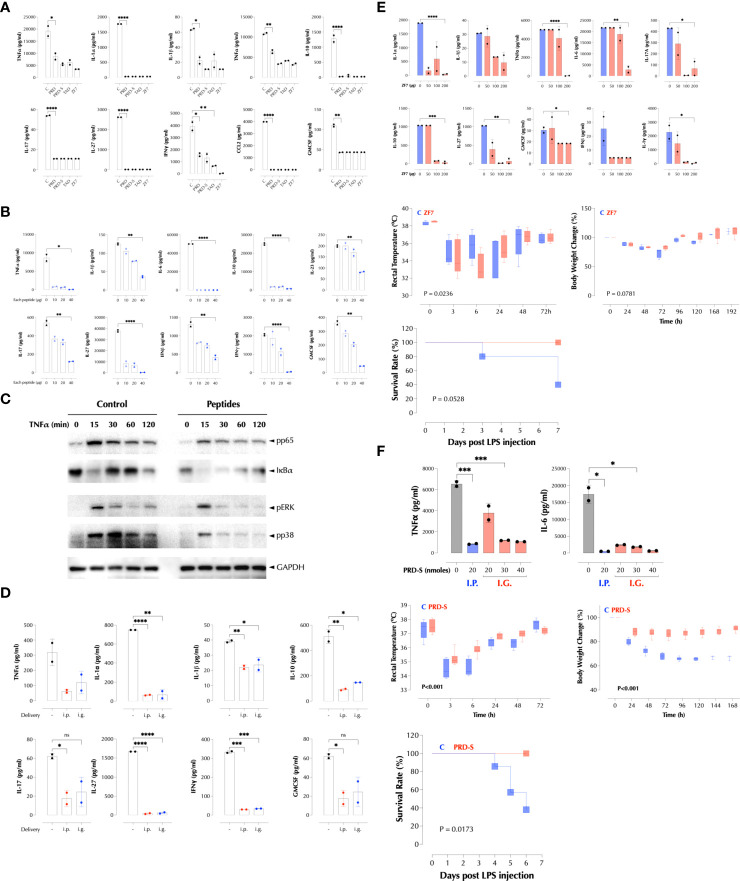
PRD, TAD, and ZF7 peptides protect mice from endotoxic injury. **(A)** PRD, TAD, and ZF7 peptides inhibit the cytokine production by LPS in a dose-dependent manner. C57Bl/6 (n=2) mice were injected i.p with LPS (50µg/mouse) and 20nmoles of each peptide at the same time. The serum cytokine levels were measured 2h post injection by LegendPlex. **(B)** The combination of PRD, TAD, and ZF7 peptides inhibits the cytokine production by LPS. C57Bl/6 (n=2) mice were injected i.p with LPS (50µg/mouse) and the indicated amount of each peptide at the same time. The serum cytokine levels were measured 2h post injection by LegendPlex. **(C)** A combination of PRD, TAD, and ZF7 peptides inhibit the TNF signaling. HCT-8 cells were treated with the peptide combination (2µg each peptide/ml) for 30min and stimulated with TNFα (2ng/ml) as indicated. Activation of NF-κB and MAP kinases was measured by western blotting. **(D)** A combination of PRD, TAD, and ZF7 peptides (40µg each peptide) inhibits the cytokine production by LPS when administered by gavage. C57Bl/6 (n=2) mice were injected with the peptides i.p or i.g. (intragastric) 30min before LPS (50µg/mouse) injection. The serum cytokine levels were measured 2h post injection by LegendPlex. **(E)** ZF7 peptide protects mice from the LPS-induced cytokines, symptoms, and lethality. Top, C57Bl/6 (n=2) mice were injected i.p with LPS (200µg/mouse) and the indicated amount of ZF7 peptide, the serum cytokine levels were measured 2h post injection by LegendPlex. Middle, C57Bl/6 (n=6) mice were injected i.p with LPS (200µg/mouse) or LPS + ZF7 peptide (300µg/mouse), and rectal temperature and body weight were measured at the indicated time points. (Statistics, 2-way ANOVA) Bottom, C57Bl/6 (n=6) mice were injected i.p with LPS (200µg/mouse) or LPS + ZF7 peptide (300µg/mouse), and the mortality was monitored for 7 days. (P = 0.0528 by Kaplan-Meier analysis) **(F)** Oral delivery of PRD-S protects mice from the LPS-induced cytokines, symptoms, and lethality. Top: C57Bl/6 (n=2) mice were injected i.p with LPS (200µg/mouse) and i.p or by gavage the indicated amount of PRD-S peptide (30min before LPS injection), the serum cytokine levels were measured 2h post injection by ELISA. Middle: C57Bl/6 (n=7) mice were injected i.p with LPS (200µg/mouse) or LPS + PRD-S peptide (30nmoles/mouse). PRD-S was administered twice by gavage 30min before and 48h after LPS injection, and the rectal temperature and body weight changes were measured at the indicated time points. (Statistics, 2-way ANOVA) Bottom: The mortality was monitored for 7 days. (P = 0.0528 by Kaplan-Meier analysis). * P < 0.05, ** P < 0.01, *** P < 0.001, ****: 0.0001, ns, not significant.

We next tested if these peptides inhibit LPS response in mice as they inhibited it in RAW cells (**Supplementary Figure 4I**). When delivered with LPS at the same time, the peptides significantly decreased LPS-induced cytokines in the serum, either individually (**Figure 7A**) or together (**Figure 7B**). As expected, the peptides together inhibited TNFα-induced proximal signaling pathways (**Figure 7C**), consistent with the broad inhibition on the downstream genes. The combined peptides were also effective when administered by gavage (**Figure 7D**). Next, we tested if ZF7 peptide can protect mice from a lethal dose of LPS (200µg/mouse). ZF7 inhibited LPS-induced production of cytokines in a dose-dependent manner (**Figure 7E**, top). When co-administered with LPS, ZF7 significantly mitigated the LPS-induced symptoms such as hypothermia and body weight loss (**Figure 7E**, middle), and a similar data were obtained with the combination of 3 peptides (data not shown). ZF7 also protected mice from LPS-induced mortality (**Figure 7E**, bottom). We next tested if oral delivery of PRD-S can protect LPS-induced inflammation in mice. Indeed, PRD-S also inhibited LPS-induced TNFα and IL-6 expression in mice when administered i.p. or by gavage (i.g.) (**Figure 7F**, top). To test its efficacy against LPS-induced inflammation, PRD-S (30nmoles) was administered twice by gavage 30min before and also 2 days after i.p injection of a lethal dose of LPS (200µg/mouse). PRD-S significantly inhibited hypothermia and body weight loss (**Figure 7F**, middle), and completely protected mice from LPS-induced lethality (**Figure 7F**, bottom). All control mice developed severe bloody diarrhea after 48 hours after LPS injection while 3 of PRD-S-treated mice had a trace of blood in the loose stool. These data provide a proof of concept that a new class of anti-inflammatory peptides may be effective against inflammatory diseases such as autoimmunity and acute infections.

## Discussion

Attenuation of inflammatory responses is essential to maintain the integrity of tissues and organisms and thus a variety of mechanisms are put in place intracellularly (negative regulators of signal transduction such as A20) and extracellularly (anti-inflammatory cytokines such as IL-10). Cellular responses to inflammatory stimuli are also influenced by the microenvironmental factors such as mechanical forces. The Hippo signaling pathway is activated by various mechanical cues, inhibiting the transcriptional activity of the YAP/TAZ/TEAD triad (4). How mechanical forces and the Hippo signaling influence inflammatory responses has not been explored extensively. We discovered that inflammatory responses to TNF are suppressed by cytoskeletal tension and thus explored the mechanism behind this phenomenon.

Inflammatory responses involve tissue damages and breakdown of extracellular matrix by degrative enzymes and cytotoxic substances, which can reduce cytoskeletal tension. As the inflammatory responses are significantly elevated when cells are under a low cytoskeletal tension, we speculate that the inflammatory responses to TNF, IL-1, or LPS are amplified in diseased tissues such as synovial tissues of arthritis patients or colonic tissues of IBD (inflammatory bowel disease) patients, probably exacerbating chronicity of these diseases. On the other hand, during the resolution phase of inflammation or carcinogenesis, fibrosis or EMT (epithelial-to-mesenchymal transition) can occur respectively, which may increase the cytoskeletal tension to cause attenuation of inflammatory responses. Therefore, the impact of mechanical cues should be taken into an account when the inflammatory responses are measured.

A20 (TNFAIP3) is essential to maintain immune homeostasis as loss of function mutation in humans or mice leads to autoinflammation or cancer (13, 14, 19, 20). Although originally the ubiquitin-editing function of A20 was thought to be essential for its suppressive function, recent findings demonstrated that its ubiquitin-binding domain, ZF7 particularly, controls the suppressive activity. A20 protein levels are relatively low under the steady-state condition but it is induced by the inflammatory stimuli, making it a feedback inhibitor. Besides the temporal regulation of A20 expression, how ZF7 exactly inhibits the inflammatory signaling is not clear. Our data shed new insights on how A20 functions to suppress inflammatory signals in collaboration with YAP9.

Our data uncovered a new negative regulator in the inflammatory signal transduction activated by TNF, IL-1, and LPS. Because of the difference in the mouse and human YAP gene, the *in vivo* validation of our findings in mice is not feasible by a knockout strategy. A knock-in of human YAP2 or YAP9 in mice should provide an answer. Humans are more sensitive to LPS than mice by several orders of magnitude (21) and we speculate the difference in YAP gene structure may explain at least partially. Further investigation on the A20/YAP9 axis may uncover the answer to this question and provide new therapeutic opportunities.

Biologics including TNF antagonists transformed the treatments of chronic inflammatory diseases such as RA and IBD in the recent decades. However, a high percentage of patients do not benefit from these drugs for various reasons and some responders develop resistance. One of new strategies to treat these diseases would be targeting downstream signaling molecules shared by the inflammatory receptors such as TNFR and IL-1R. Our data show that PRD, TAD, and ZF7 peptides inhibit the signaling by these receptors by binding to molecules such as RIP and TAK1. As the signaling complexes are composed of a number of proteins, which molecule each peptide directly binds to needs to be further characterized. In conclusion, we have discovered an anti-inflammatory pathway and anti-inflammatory peptides that may provide a new pathway to treatments of inflammatory diseases.

## Materials and methods

### Reagents

The reagents (**Table 1**) and antibodies (**Table 2**) used in the study are as follows.

**Table 1:** Reagents.

**NAME**	**Cat. No.**	**Source**
TNFα	300-03A	PEPROTECH
LPS	M9524	AbMole
IL-1β	200-01B-2	PEPROTECH
Vitrogel	TWG001	TheWall
Cytochalasin D	PHZ1063	ThermoFisher
Phalloidin	CA1610	Solarbio
DAPI	C0065	Solarbio
Luciferase assay kit	E1910	Promega
Streptavidin magnetic beads	5947	Cell Signaling Technology

**Table 2:** Antibodies.

	**Fluorescent label**	**Clone Number**	**Source**
Human YAP1	Alexa Fluor 488	17A2	Cell Signaling Technology
Human CD3	APC/Cy7	HIT3a	BioLegend
Human CD14	Alexa Fluor 488	HCD14	BioLegend
Myc		9B11	Cell Signaling Technology
HA		C29F4	Cell Signaling Technology
β-Tublin		9F3	Cell Signaling Technology
IκBα		L35A5	Cell Signaling Technology
p-p38		D3F9	Cell Signaling Technology
p-ERK		D13.14.4E	Cell Signaling Technology
p-p65		S536	Cell Signaling Technology
YAP1		D8H1X	Cell Signaling Technology
TNFR1		C25C1	Cell Signaling Technology
TAK1		D94D7	Cell Signaling Technology
RIP		D94C12	Cell Signaling Technology
GAPDH		1A6	Bioworld
Mouse TNFα	Alexa Fluor 488	MP6-XT22	BioLegend

### Antibodies

Antibodies used in this study are listed in **Table 2**.

The following reagents (**Table 3**) were used for RNA isolation and qPCR.

**Table 3:** Reagents for RNA Isolation and qPCR.

Name	Catalog No.	source
ChamQ Universal SYBR qPCR Master Mix	Q711-02	Vazyme
Hieff UNICON qPCR SYBR Green Master Mix	11198ES03	Yeasen
HiScript III RT SuperMix for qPCR	R323-01	Vazyme
NucleoZOL	740404.200	TaKaRa Bio Inc.

### siRNA

All double-stranded siRNAs (**Table 4**) against Mus musculus/Homo Sapiens genes were designed and purchased from Tsingke Biological Technology (China).

**Table 4:** siRNAs.

**Gene name**	**Sense Sequence**
Human YAP1	5’- GACAUCUUCUGGUCAGAGATT-3’
Human A20	5’-AAGCCUGCCUCCAGGAUGUUATT -3’
Mouse YAP1	5’- AACCAGAGGAUCACUCAGAGU-3’

### Plasmids

All expression constructs in **Table 5** were chemically synthesized, cloned, and sequenced.

**Table 5:** Plasmids.

**Insert**	**Vector**	**Tag**
Human YAP2	pcDNA3.1(+)	HA
Human YAP9	pcDNA3.1(+)	HA
Human A20	pcDNA3.1(+)	Myc
Human RIP	pcDNA3.1(+)	HA
Human TAK1	pcDNA3.1(+)	Myc
Human TNFR1	pcDNA3.1(+)	HA

YAP2 phosphorylation site mutants were a generous gift from Kun-Liang Guan (22). The YAP1/TAZ reporter construct was previously described (5).

### Cell culture

Cells were routinely maintained in cell culture incubators (95% air, 5% CO2 in gas phase, at 37°C). FLS (human fibroblast-like synoviocytes) and HCT-8 cells were purchased from American Type Culture Collection and cultured in DMEM medium from Gibco, supplemented with 10% heat-inactivated fetal bovine serum (Biological Industries, USA), 100U/mL penicillin and 100μg/mL streptomycin (ThermoFisher Scientific). All cultures were routinely tested for Mycoplasma contamination.

### 3D cell culture

For 3D Matrigel cultures, 50 μl of cold complete medium containing 4 × 10^4^ cells was added to 25 μl of cold Matrigel, gently mixed with ice-cold pipette tips and deposited into either a 8-well chambered coverglass (Nalge Nunc International) or a 48-well plate (Corning Incorporated) depending on the experiment and incubated at 37 °C for 30 min. For some experiments, 3D cultures were scaled to 24-well plates. For Vitrogel culture, cells were resuspended in 250μl Vitrogel diluted 1:2 in 0.5% PBS at 1×10^6^/ml for 24-well plate, and complete media was added when gel solidifies. Cells were then covered with complete medium when gel solidifies and grown at 37°C under 5% CO2, changing medium every 2 days.

### Peptide synthesis and transfection

Peptides were synthesized and the sequences were verified by Nanjing GenScript Biotech Co., Ltd (Nanjing, China). Peptides were transfected into the cells using Pierce Protein Transfection Reagent Kit™ according to the manufacturer’s instruction. The Pierce Reagent in methanol was pipetted into a tube and the solvent was evaporated in the hood. Peptides were diluted in PBS and mixed with the dried Pierce Reagent. The peptides/Reagent complex was added to the cells in serum-free medium. After 4 hours, the medium containing serum was added to the cells and the cells were used for experiments.

### Lentivirus production and infection

Lentiviruses were packaged by GenePharma Inc. (Shanghai, China) and cells were infected with the viruses in the presence of polybrene (5µg/ml) overnight. Some stable cell lines were generated by selection with puromycin (5µg/ml).

### Animals

All animal work was done following the Guide for the Care and Use of Laboratory Animal and approved by the institutional ethics committee of School of Basic Medical Sciences of Guangzhou Medical University, in accordance with the vivarium of Institute of Animal Health, Guangdong Academy of Agricultural Sciences. All efforts were made to minimize the number of animals used and their suffering. All mice were housed in specific pathogen-free conditions before use. Male and female mice were 6–8 weeks old at the time of use. C57BL mice were obtained from Jinan Pengyue Animal Center, and SKG mice were obtained from CLEA Japan, Inc. BALC/c and Rag2^-/-^ mice were purchased from GemPharmatech Co., Ltd. (China).

### Gene knockout in cells by Crispr/Cas9

For human YAP and A20 knockout, a Crispr/Cas9 construct in LGE-4(LentiV2-gRNACas9Puro) containing the sgRNA (**Table 6**) was packaged in Lentiviruses (GenePharma, China). HCT-8 cells were infected with either control viruses or YAP-KO/A20-KO viruses and selected with puromycin. KO was confirmed by qPCR.

**Table 6:** Short guide RNAs (sgRNAs).

Gene	short guide RNA (5’-3’)
Human YAP1	sgRNA#1: CCCTGCGGGGGCTGCGAAGG sgRNA#2: ACCCGGGCAACCGGCACCCG
Human A20	GAAGCTCAGAATCAGAGATT

### Isolation of human PBMCs

For human PBMCs and T cell isolation, the study was approved by the institutional ethics committee of School of Basic Medical Sciences of Guangzhou Medical University and a signed informed consent was obtained from every participant before being included into the study. blood was drawn into heparin sodium-containing tubes (K2E Vacutainer, BD) from healthy volunteers in the laboratory (non-shipped). Samples were processed within 2 hours. The time interval between the two blood drawings of each individual was 20–30 days and none of the individuals showed or reported any disease symptoms. All steps of PBMC preparation were carried out at room temperature. The blood from the same donor was pooled and mixed 1:1 with pre-warmed (room temperature) 1×PBS (without Ca^2+^ or Mg^2+^). The mixture was layered onto Lymphoprep (Stem Cell Technologies) in a SepMate™ tube and centrifuged at 1200rcf for 10min. The top layer containing enriched mononuclear cells was poured into a new tube and washed with PBS containing 2% FBS twice by centrifuging at 300rcf for 8min. Cells were resuspended in RPMI1640 medium for subsequent procedures.

### Western blotting (WB) and immunoprecipitation (IP)

All antibodies used in WB were purchased from Cell Signaling Technology. IP was performed by adding the indicated antibody and protein A-beads to the cell lysates.

### Pull-down assay of biotinylated peptides

HCT-8 cells were cultured on a 10cm petri-dish, and cell lysates were collected with protein lysis buffer (Cell Signaling Technology) when cell confluency reaches70%-80%. The biotinylated PRD, TAD or ZF7 peptides were incubated with lysates and then streptavidin magnetic beads (Cell Signaling Technology) were used to pull down biotinylated peptides together with proteins binding to them. Then the beads were washed with lysis buffer thoroughly before loaded with SDS-PAGE protein loading buffer to 4-20% gradient SDS-PAGE gels. The membranes were then probed with different antibodies.

### Nucleic acid extraction and RNA reverse transcription

Total RNAs were extracted using Trizol (ThermoFisher Scientific) or NucleoZOL (TaKaRa) according to the manufacturer’s protocol. Extracted RNA was dissolved in RNase- and DNase-treated water and then immediately reverse-transcribed using HiScript Q RT SuperMix for qPCR (Vazyme). cDNA was stored at −20°C for subsequent qPCR analyses.

### qPCR primers

All qPCR primers used in the study are listed in **Table 7**.

**Table 7:** qPCR Primers.

Gene	Forward Primer (5’-3’)	Reverse Primer (5’-3’)
Human GAPDH	ATCACCATCTTCCA GGAGCGAG	GGGCAGAGATGATGAC CCTTTTG
Human CTGF	AGGAGTGGGTGTG TGACGA	CCAGGCAGTTGGCTCTA ATC
Human ANKRD1	AGTAGAGGAACTG GTCACTGG	TGGGCTAGAAGTGTCTT CAGAT
Human IκBα	AAGTGATCCGCCA GGTGAAG	CTGCTCACAGGCAAGGT GTA
Human MMP1	GACATTGATGGCA TCCAAGC	GTCGGCAAATTCGTAAG CAG
Human MMP3	AGTCTTCCAATCC TACTGTTGCT	TCCCCGTCACCTCCAAT CC
Human BMP	ACACATGTCACAT TGCAACCC	GTAAAACGACGGCCAG TAGGGAAAGCAGTTCA GGGAG
Human YAP1	CCCTCGTTTTGCC ATGAAC	GGCCTCTGCTGTAGTCC TTT
Human A20	GCCTCCAGGATGT TACCAGG	GGCCTCTGCTGTAGTCC TTT
Human IL-6	ACTCACCTCTTCA GAACGAATTG	CCATCTTTGGAAGGTTC AGGTTG
Human IL-8	CTTGGCAGCCTT CCTGATTT	TTCCTTGGGGTCCAGAC AGA
Human IL-23A	CTCAGGGACAAC AGTCAGTTC	ACAGGGCTATCAGGGA GCA
Human IL-32	GAAGGTCCTCTC TGATGACA	AAGTAGAGGAGTGAGCT CTG
Human CCL-2	GCAATCAATGCC CCAGTCAC	TGCTTGTCCAGGTGGTC CAT
Human CXCL1	AGCTCTTCCGCT CCTCTCAC	AGTGTGGCTATGACTTC GGTT
Human CXCL2	CGGGAGTTACGC AAGACAG	ATGGTTGGGGCTGGAA AG
Human CXCL3	CCAAACCGAAG TCATAGCCAC	TGCTCCCCTTGTTCAGT ATCT
Human CXCL5	AGCTGCGTTGC GTTTGTTTAC	TGGCGAACACTTGCAGA TTAC
Human CXCL10	GTGGCATTCAAGGAGTACCTC	TGATGGCCTTCGATTCT GGATT
Human CXCL11	GACGCTGTCTTTGCATAGGC	GGATTTAGGCATCGTTG TCCTTT
Human CCL-20	CAACTTTGACTGCTGTCTTGGATA	TTGACTTTTTTACTGAG GAGACGC
Human MMP3	AGTCTTCCAATCCTACTGTTGCT	TCCCCGTCACCTCCAAT CC
Human BMP2	CCCAGCGTGAAAAGAGAGAC	CCATGGTCGACCTTTAG GAG
Human OMD	CTCCAAACTGCAGACAAATGC	GTAAAACGACGGCCAG TGCAGATTTCTGCCAT TCCC
Human OPG	GCTAACCTCACCTTCGAG	TGATTGGACCTGGTT ACC
Human COX-2	TAAGTGCGATTGTACCCGGAC	TTTGTAGCCATAGTCAG CATTGT
Human PTGS1	CGGTGAAACTCTGGCTAGACAG	GCAAACCGTAGATGCTC AGGGA
Mouse YAP1	GAAGGAGAGACTGCGGTTGA	TGCGCAGAGCTAATTCC TGA

### ELISA

Human IL-8 ELISA kit was purchased from ThermoFisher and ELISA was performed according to the manufacturer’s instruction. Human CCL-20 ELISA kit was purchased from BioLegend and ELISA was performed according to the manufacturer’s instruction.

### Transfection of suspension cells by electroporation

For gene knockdown or overexpression in suspension cells, siRNAs and plasmids were introduced into cells by Neon Transfection System (Invitrogen) according to the manufacturer’s protocol. After the cells were pre-incubated in complete cell culture medium containing the nucleic acid transfection enhancer NATE (1%) for 30min, the electroporation pipette tip was filled with 2×10^6^ cells in transfection T buffer containing siRNA/plasmids for primary cells and R buffer containing siRNA/plasmids for cell lines. Three milliliters of Electrolytic buffer were added to a buffer container. The electrical conditions were set according to Neon transfection cell database. After electroporation, cells were rapidly dispensed into 5ml round bottom sterile tubes or cell culture plates using a pipette, and were then cultured in a CO_2_ incubator at 37°C.

### siRNA transfection with RNAiMAX

Cells are transfected using Lipofectamine RNAiMAX (Life Technologies, Grand Island, NY). The day prior to transfections, cells are plated on cell-culture plates (Corning) at 500 µL/well for 12-well plates and 1ml/well for 6-well plates at density of 4 × 10^5^–5 × 10^5^ cells/mL in the indicated growth medium and propagated to 80% confluency at the time of transfection. Transfection was performed according to the manufacturer’s instruction.

### FACS analysis

To measure YAP1 expression, cells were fixed and permeabilized using CytoFix/CytoPerm buffers (BD Biosciences) according to the manufacturer’s instruction. Cells were stained with anti-YAP1 antibody-FITC (Cell Signaling Technology) and measured by flow cytometry (FACSVerse, BD Biosciences). Human PBMCs were stained with anti-CD14 antibody-PE and anti-CD3 antibody-APC before being fixed and permeabilized.

### Immunofluorescence confocal microscopy

HCT-8 or FLS cells were seeded in chambered coverglasses and different treatments were applied according to experimental designs. Before analysis, the cells were fixed and permeabilized using CytoFix/CytoPerm kit (BD Biosciences), and stained with anti-YAP fluorescent antibody, phalloidin and DAPI, and then washed in Perm/Wash buffer. For peptide localization, HCT-8 cells were incubated with biotinylated peptides, and then fixed and permeabilized using CytoFix/CytoPerm kit, and stained with streptavidin Alexa fluor-647 antibody, phalloidin and DAPI. Frozen mouse colon tissues were, air-dried, fixed with 4% PFA, permeabilized with PBS-T, blocked with 2% rat serum in PBS for 30min, and then stained with anti-CD4-AF (Alexa Fluor) 647 (BioLegend), anti-IL-17A-AF488 (ThermoFisher) and DAPI. All samples were visualized under Zeiss LSM 880 confocal microscope.

### RNA sequencing

Total RNA was extracted using TRIzol (ThermoFisher Scientific). RNAseq libraries were prepared using the VAHTS TM mRNAseq V2 Library Prep Kit for Illumina^®^ (Vazyme #NR601), and libraries were sequenced on an Illumina Xten to a read depth of 25–35 million reads per sample. Sequencing quality was assessed using Agilent 4200 Bioanalyzer. The sequencing data was filtered with SOAPnuke (v1.5.2) to remove the reads containing the sequencing adapter; Reads whose low-quality base ratio (base quality less than or equal to 5) was more than 20% were removed; After removing reads whose unknown base (‘N’ base) ratio is more than 5%, the clean reads were obtained and stored in FASTQ format. The clean reads were mapped to the reference genome using HISAT2 (v2.0.4), and then expression level of gene was calculated by STRING TIE (v1.2.3). To get an insight to the change of phenotype, GO (http://www.geneontology.org/) and KEGG (https://www.kegg.jp/) enrichment analysis of annotated different expressed gene was performed by Phyper based on Hypergeometric test. The significant levels of terms and pathways were corrected by P value with a rigorous threshold (P value ≤ 0.05) by Bonferroni.

### Legendplex™

To simultaneously quantify the concentration of the soluble inflammatory cytokines and chemokines, the cell culture supernatant or mouse sera were analyzed by using the bead-based multiplex LEGENDplex™ (Biolegend) according to the manufacturer’s instruction.

### LPS study in mice

All experiments were approved by the animal ethics committee of Guangzhou Medical University and carried out in accordance with established Guiding Principles for Animal Research. Female C57 mice (20-25g) were injected with bacterial endotoxin LPS (Escherichia coli, serotype O55:B5; 10 mg/kg, i.p.) alone or together with the ZF7 peptide (Control animals received sterile PBS. Animals were killed by anesthetic overdose 4 hrs after LPS/PBS injection. Blood was collected by reto-orbital bleeding into tubes 3h post injection for legendplex analysis, and for mortality monitor, mouse rectal temperature was measured at indicated time points after injection, while mortality was recorded daily.

## Data availability statement

The datasets presented in this study are deposited in the GEO repository, accession number GSE203438.

## Ethics statement

The studies involving human participants were reviewed and approved by Guangzhou Medical University. The patients/participants provided their written informed consent to participate in this study. The animal study was reviewed and approved by Guangzhou Medical University.

## Author contributions

All authors contributed significantly to the drafting and editing of this manuscript. JL and FY conceived the manuscript idea and revised the manuscript content. FY, LS, DF, YB, and BL performed experiments. All authors have read and agreed to the final version of the manuscript.

## Funding

This work was supported by Guangzhou Medical University Startup Fund to JL.

## Conflict of interest

The authors declare that the research was conducted in the absence of any commercial or financial relationships that could be construed as a potential conflict of interest.

## Publisher’s note

All claims expressed in this article are solely those of the authors and do not necessarily represent those of their affiliated organizations, or those of the publisher, the editors and the reviewers. Any product that may be evaluated in this article, or claim that may be made by its manufacturer, is not guaranteed or endorsed by the publisher.
