# Supplementary material for: YAP9/A20 complex suppresses proinflammatory responses and provides novel anti-inflammatory therapeutic potentials
Source: Front Immunol. 2022 Aug 15;13:914381. doi: 10.3389/fimmu.2022.914381 (PMC9420849; doi:10.3389/fimmu.2022.914381)

Supplemental Data

Supplemental Figure 1. Cell culture matrix regulates the TNF response.

A. Enhanced TNF response in 3D FLS is evident within 2 days of culture. FLS in 2D or 3D (Vitrogel) were stimulated at the indicated time points, and the mRNA levels were measured by qPCR. The results are the representative of several similar experiments. **B.** TNF response is enhanced in different 3D matrices. FLS were plated in 2D, Matrigel or Vitrogel stimulated with TNFα (1ng/ml) for 2h. The gene expression levels were measure by qPCR. (Statistics by Student T test, *: $P < 0.05$, **: $P < 0.01$, ***: $P < 0.001$, ****: $P < 0.0001$) **C.** TNF response is enhanced in 3D compared to 2D HCT-8 cells. HCT-8 cells were plated in plastic culture plate (2D) or Vitrogel (3D) and stimulated with TNFα (2ng/ml) as indicated, and the gene expression levels were measure by qPCR. (Statistics by 2-way ANOVA)

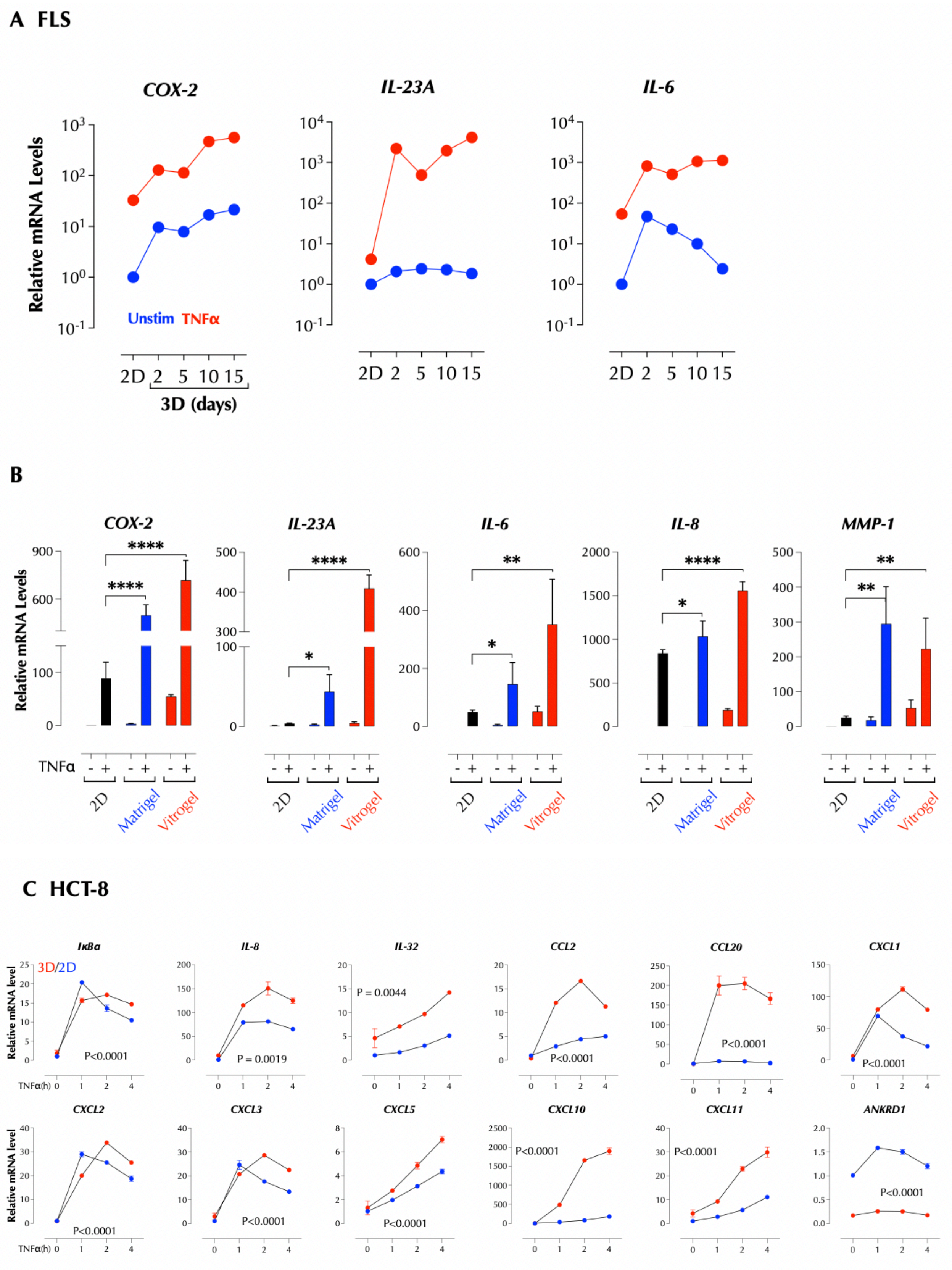
A FLS
COX-2
IL-23A
IL-6
Relative mRNA Levels
Unstim TNFα
2D 2 5 10 15
3D (days)
B
COX-2
IL-23A
IL-6
IL-8
MMP-1
Relative mRNA Levels
****
**
*
TNFα
- +
2D
Matrigel
Vitrogel
C HCT-8
IκBα
IL-8
IL-32
CCL2
CCL20
CXCL1
3D/2D
Relative mRNA level
P<0.0001
P = 0.0019
P = 0.0044
TNFα(h) 0 1 2 4
CXCL2
CXCL3
CXCL5
CXCL10
CXCL11
ANKRD1

Supplemental Figure 2. YAP1 and the Hippo signaling mediate the cytoskeletal tension-induced suppression of the TNF response.

A. YAP1 does not regulate F-actin formation. YAP1 and F-actin were visualized in Control and YAP-1-KD FLS by confocal. **B.** YAP1 is responsible for the suppression of the TNF response by cytoskeletal tension in 2D culture. Control and YAP1-KO FLS were treated with Cyto D (1µg/ml) for 2h and stimulated with TNFα (1ng/ml) overnight. The IL-8 levels were measured by ELISA. (*: $P < 0.05$, **: $P < 0.01$, ns: not significant)

A

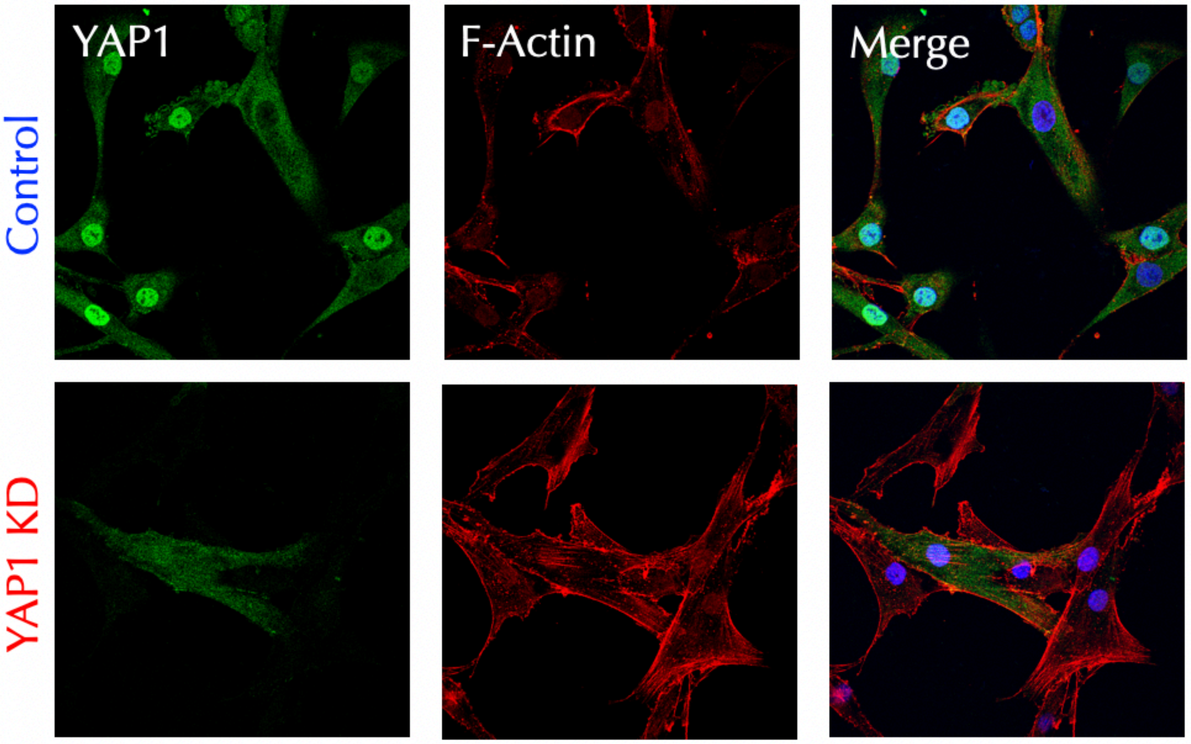
YAP1
F-Actin
Merge
Control
YAP1 KD

B

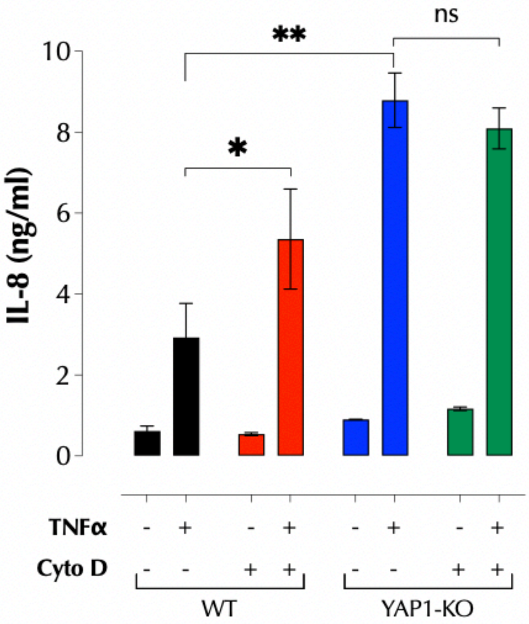
IL-8 (ng/ml)
10
8
6
4
2
0
**
ns
*
TNFα
Cyto D
WT
YAP1-KO

Supplementary Figure 3. Cytoskeletal tension regulates differential expression YAP1 isoforms. **A.** Design of the PCR primers to detect different YAP1 isoforms. **B.** Non-quantitative RT-PCR of YAP1 isoforms (40 cycles). One set (blue) amplifies all isoforms and the other (red) only the isoforms with the full TAD sequence. **C.** In 3D culture of FLS, the total YAP1 mRNA is higher than 2D FLS but the isoforms lacking the full TAD sequence are dominant. (**: $P < 0.01$, ***: $P < 0.001$, ****: $P < 0.0001$) **D.** CytoD increases the total YAP1 mRNA level but depresses the isoforms containing the full TAD sequence. 2D FLS were treated with the CytoD (1µg/ml) for the indicated periods and qPCR was performed. **E.** Mouse YAP1 lacks the PRD. The protein sequences of human YAP9 and mouse YAP isoforms were aligned using Clustal Omega. **F.** YAP1 depletion in the mouse macrophage cell line RAW264.7 increases the basal TNF level but decreases LPS-induced TNF expression. The results are the representative of two experiments.

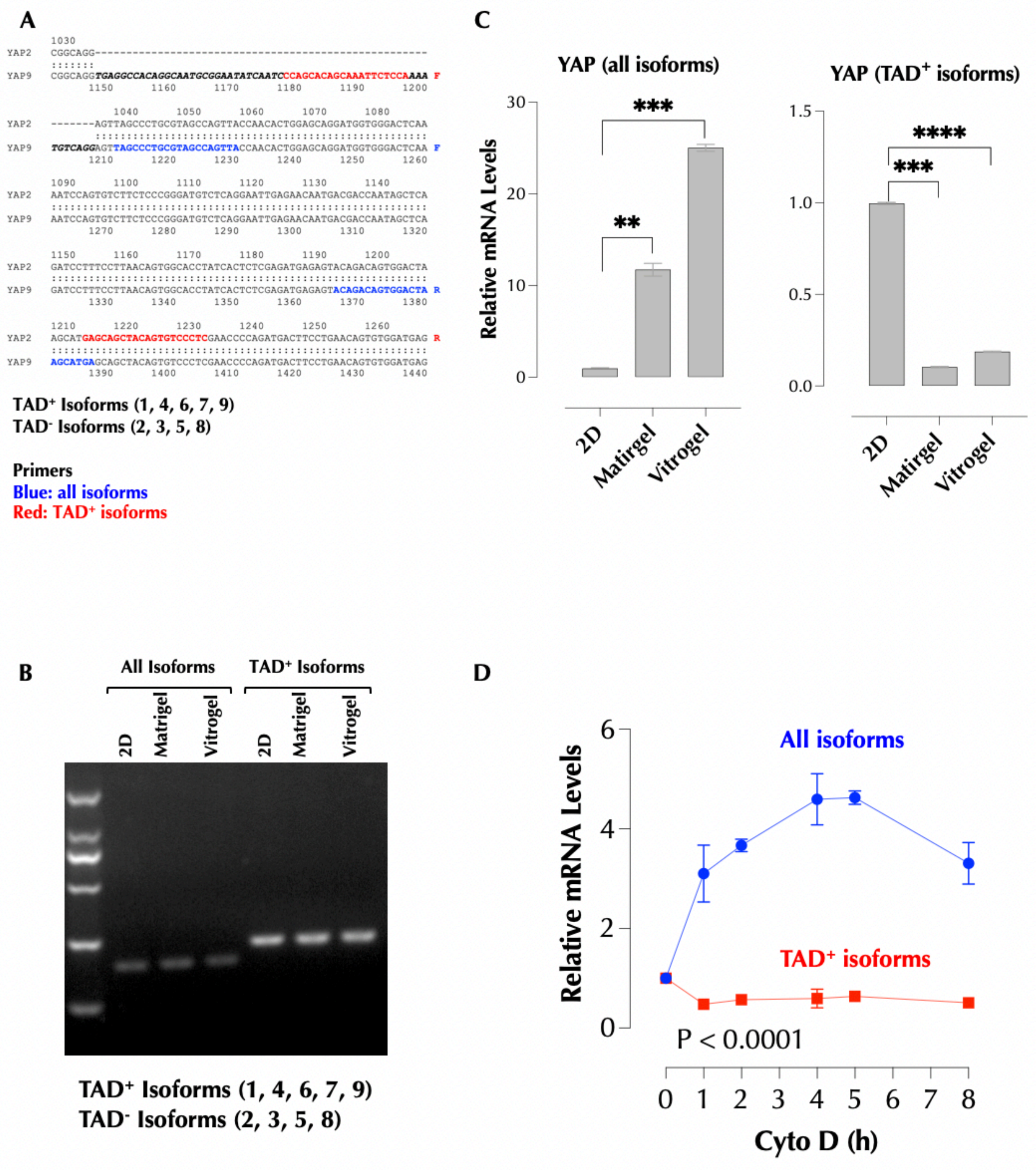
A
YAP2
YAP9
TAD+ Isoforms (1, 4, 6, 7, 9)
TAD- Isoforms (2, 3, 5, 8)
Primers
Blue: all isoforms
Red: TAD+ isoforms
B
All Isoforms
TAD+ Isoforms
2D
Matrigel
Vitrogel
TAD+ Isoforms (1, 4, 6, 7, 9)
TAD- Isoforms (2, 3, 5, 8)
C
YAP (all isoforms)
YAP (TAD+ isoforms)
Relative mRNA Levels
2D
Matrigel
Vitrogel
D
All isoforms
TAD+ isoforms
Relative mRNA Levels
P < 0.0001
Cyto D (h)

E

hYAP9 vs. mYAP isoforms

```
NP_001269030.1    MDPGQQPPPQPAPQGQGQPPSQPPQGQGPPSGPGQPAPAATQAAPQAPPAGHQIVHVRGD    60
XP_006509920.1    MEPAQQPPPQPAPQGPAPPSV---------------SPAGTPAAPPAPPAGHQVVHVRGD    45
XP_006509919.1    MEPAQQPPPQPAPQGPAPPSV---------------SPAGTPAAPPAPPAGHQVVHVRGD    45
XP_006509918.1    MEPAQQPPPQPAPQGPAPPSV---------------SPAGTPAAPPAPPAGHQVVHVRGD    45
XP_006509917.1    MEPAQQPPPQPAPQGPAPPSV---------------SPAGTPAAPPAPPAGHQVVHVRGD    45
XP_006509915.1    MEPAQQPPPQPAPQGPAPPSV---------------SPAGTPAAPPAPPAGHQVVHVRGD    45
XP_006509916.1    MEPAQQPPPQPAPQGPAPPSV---------------SPAGTPAAPPAPPAGHQVVHVRGD    45
                  *:*.***********  .  *               :**.* *** *******:******

NP_001269030.1    SETDLEALFNAVMNPKTANVPQTVPMRLRKLPDSFFKPPEPKSHSRQASTDAGTAGALTP    120
XP_006509920.1    SETDLEALFNAVMNPKTANVPQTVPMRLRKLPDSFFKPPEPKSHSRQASTDAGTAGALTP    105
XP_006509919.1    SETDLEALFNAVMNPKTANVPQTVPMRLRKLPDSFFKPPEPKSHSRQASTDAGTAGALTP    105
XP_006509918.1    SETDLEALFNAVMNPKTANVPQTVPMRLRKLPDSFFKPPEPKSHSRQASTDAGTAGALTP    105
XP_006509917.1    SETDLEALFNAVMNPKTANVPQTVPMRLRKLPDSFFKPPEPKSHSRQASTDAGTAGALTP    105
XP_006509915.1    SETDLEALFNAVMNPKTANVPQTVPMRLRKLPDSFFKPPEPKSHSRQASTDAGTAGALTP    105
XP_006509916.1    SETDLEALFNAVMNPKTANVPQTVPMRLRKLPDSFFKPPEPKSHSRQASTDAGTAGALTP    105
                  ************************************************************

NP_001269030.1    QHVRAHSSPASLQLGAVSPGTLTPTGVVSGPAATPTAQHLRQSSFEIPDDVPLPAGWEMA    180
XP_006509920.1    QHVRAHSSPASLQLGAVSPGTLTASGVVSGPAAAPAAQHLRQSSFEIPDDVPLPAGWEMA    165
XP_006509919.1    QHVRAHSSPASLQLGAVSPGTLTASGVVSGPAAAPAAQHLRQSSFEIPDDVPLPAGWEMA    165
XP_006509918.1    QHVRAHSSPASLQLGAVSPGTLTASGVVSGPAAAPAAQHLRQSSFEIPDDVPLPAGWEMA    165
XP_006509917.1    QHVRAHSSPASLQLGAVSPGTLTASGVVSGPAAAPAAQHLRQSSFEIPDDVPLPAGWEMA    165
XP_006509915.1    QHVRAHSSPASLQLGAVSPGTLTASGVVSGPAAAPAAQHLRQSSFEIPDDVPLPAGWEMA    165
XP_006509916.1    QHVRAHSSPASLQLGAVSPGTLTASGVVSGPAAAPAAQHLRQSSFEIPDDVPLPAGWEMA    165
                  *********************** :********:*:************************

NP_001269030.1    KTSSGQRYFLNHIDQTTTWQDPRKAMLSQMNVTAPTSPPVQQNMMNSASGPLPDGWEQAM    240
XP_006509920.1    KTSSGQRYFLNHNDQTTTWQDPRKAMLSQLNVPAPASPAVPQTLMNSASGPLPDGWEQAM    225
XP_006509919.1    KTSSGQRYFLNHNDQTTTWQDPRKAMLSQLNVPAPASPAVPQTLMNSASGPLPDGWEQAM    225
XP_006509918.1    KTSSGQRYFLNHNDQTTTWQDPRKAMLSQLNVPAPASPAVPQTLMNSASGPLPDGWEQAM    225
XP_006509917.1    KTSSGQRYFLNHNDQTTTWQDPRKAMLSQLNVPAPASPAVPQTLMNSASGPLPDGWEQAM    225
XP_006509915.1    KTSSGQRYFLNHNDQTTTWQDPRKAMLSQLNVPAPASPAVPQTLMNSASGPLPDGWEQAM    225
XP_006509916.1    KTSSGQRYFLNHNDQTTTWQDPRKAMLSQLNVPAPASPAVPQTLMNSASGPLPDGWEQAM    225
                  ************ ****************:** **:** * *.:****************

NP_001269030.1    TQDGEIYYINHKNKTTSWLDPRLDPRFA--MNQRISQSAPVKQPPPLAPQSPQGGVMGGS    298
XP_006509920.1    TQDGEVYYINHKNKTTSWLDPRLDPRFGKAMNQRITQSAPVKQPPPLAPQSPQGGVLGGG    285
XP_006509919.1    TQDGEVYYINHKNKTTSWLDPRLDPRFA--MNQRITQSAPVKQPPPLAPQSPQGGVLGGG    283
XP_006509918.1    TQDGEVYYINHKNKTTSWLDPRLDPRFGKAMNQRITQSAPVKQPPPLAPQSPQGGVLGGG    285
XP_006509917.1    TQDGEVYYINHKNKTTSWLDPRLDPRFGKAMNQRITQSAPVKQPPPLAPQSPQGGVLGGG    285
XP_006509915.1    TQDGEVYYINHKNKTTSWLDPRLDPRFGKAMNQRITQSAPVKQPPPLAPQSPQGGVLGGG    285
XP_006509916.1    TQDGEVYYINHKNKTTSWLDPRLDPRFA--MNQRITQSAPVKQPPPLAPQSPQGGVLGGG    283
                  *****:*********************.  *****:********************:**.

NP_001269030.1    NSNQQQQMRLQQLQMEKERLRLKQQELLRQVRPQAMRNINPSTANSPKCQELALRSQLPT    358
XP_006509920.1    SSNQQQQIQLQQLQMEKERLRLKQQELFRQ--------------------ELALRSQLPT    325
XP_006509919.1    SSNQQQQIQLQQLQMEKERLRLKQQELFRQVRPQ----------------ELALRSQLPT    327
XP_006509918.1    SSNQQQQIQLQQLQMEKERLRLKQQELFRQVRPQ----------------ELALRSQLPT    329
XP_006509917.1    SSNQQQQIQLQQLQMEKERLRLKQQELFRQ----AIRNINPSTANAPKCQELALRSQLPT    341
XP_006509915.1    SSNQQQQIQLQQLQMEKERLRLKQQELFRQVRPQAIRNINPSTANAPKCQELALRSQLPT    345
XP_006509916.1    SSNQQQQIQLQQLQMEKERLRLKQQELFRQVRPQAIRNINPSTANAPKCQELALRSQLPT    343
                  .******::******************:**                    **********

NP_001269030.1    LEQDGGTQNPVSSPGMSQELRTMTTNSSDPFLNSGTYHSRDESTDSGLSMSSYSVPRTPD    418
XP_006509920.1    LEQDGGTPNAVSSPGMSQELRTMTTNSSDPFLNSGTYHSRDESTDSGLSMSSYSIPRTPD    385
XP_006509919.1    LEQDGGTPNAVSSPGMSQELRTMTTNSSDPFLNSGTYHSRDESTDSGLSMSSYSIPRTPD    387
XP_006509918.1    LEQDGGTPNAVSSPGMSQELRTMTTNSSDPFLNSGTYHSRDESTDSGLSMSSYSIPRTPD    389
XP_006509917.1    LEQDGGTPNAVSSPGMSQELRTMTTNSSDPFLNSGTYHSRDESTDSGLSMSSYSIPRTPD    401
XP_006509915.1    LEQDGGTPNAVSSPGMSQELRTMTTNSSDPFLNSGTYHSRDESTDSGLSMSSYSIPRTPD    405
XP_006509916.1    LEQDGGTPNAVSSPGMSQELRTMTTNSSDPFLNSGTYHSRDESTDSGLSMSSYSIPRTPD    403
                  ******* * ********************************************:*****

NP_001269030.1    DFLNSVDEMDTGDTINQSTLPSQQNRFPDYLEAIPGTNVDLGTLEGDGMNIEGEELMPSL    478
XP_006509920.1    DFLNSVDEMDTGDTISQSTLPSQQSRFPDYLEALPGTNVDLGTLEGDAMNIEGEELMPSL    445
XP_006509919.1    DFLNSVDEMDTGDTISQSTLPSQQSRFPDYLEALPGTNVDLGTLEGDAMNIEGEELMPSL    447
XP_006509918.1    DFLNSVDEMDTGDTISQSTLPSQQSRFPDYLEALPGTNVDLGTLEGDAMNIEGEELMPSL    449
XP_006509917.1    DFLNSVDEMDTGDTISQSTLPSQQSRFPDYLEALPGTNVDLGTLEGDAMNIEGEELMPSL    461
XP_006509915.1    DFLNSVDEMDTGDTISQSTLPSQQSRFPDYLEALPGTNVDLGTLEGDAMNIEGEELMPSL    465
XP_006509916.1    DFLNSVDEMDTGDTISQSTLPSQQSRFPDYLEALPGTNVDLGTLEGDAMNIEGEELMPSL    463
                  ***************.********.********:*************.************

NP_001269030.1    QEALSSDILNDMESVLAATKLDKESFLTWL    508
XP_006509920.1    QEALSSEIL-DVESVLAATKLDKESFLTWL    474
XP_006509919.1    QEALSSEIL-DVESVLAATKLDKESFLTWL    476
XP_006509918.1    QEALSSEIL-DVESVLAATKLDKESFLTWL    478
XP_006509917.1    QEALSSEIL-DVESVLAATKLDKESFLTWL    490
XP_006509915.1    QEALSSEIL-DVESVLAATKLDKESFLTWL    494
XP_006509916.1    QEALSSEIL-DVESVLAATKLDKESFLTWL    492
                  ******:** *:******************
```

F

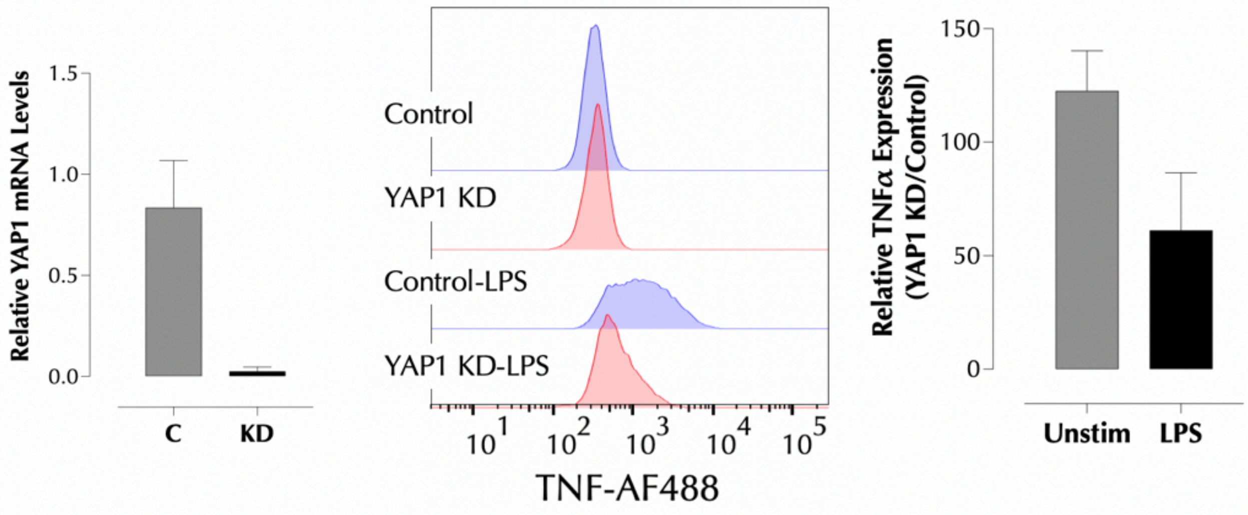
Relative YAP1 mRNA Levels
1.5
1.0
0.5
0.0
C
KD
Control
YAP1 KD
Control-LPS
YAP1 KD-LPS
10^1
10^2
10^3
10^4
10^5
TNF-AF488
Relative TNFα Expression
(YAP1 KD/Control)
150
100
50
0
Unstim
LPS

Supplemental Figure 4. YAP1 suppresses the inflammatory responses using PRD and TAD.

A. PRD and TAD peptides inhibit the TNF response. HCT-8 cells were transfected with the indicated amount PRD or TAD peptide in different amounts of the peptide transfection agent. The cells were stimulated with TNFα (2ng/ml) 4h after transfection and IL-8 was measured by ELISA the next day. **B.** Human PBMCs were transfected with PRD or TAD peptide (1µg/ml) and the inflammatory mediators were measured using LegendPlex. **C.** HCT-8 cells were stimulated with TNFα (2ng/ml) with the indicated amount of PRD-S, and IL-8 was measured by ELISA the next day. **D.** Biotinylated PRD and TAD peptides **E.** Biotinylated PRD, TAD, and ZF7 peptides penetrate cells. THP-1 cells were treated with the indicated peptide (20µg/ml) for 2h and localized by confocal microscopy. **F.** Biotinylated TAD peptide inhibits the TNF response. HCT-8 cells were stimulated with TNFα (2ng/ml) with the indicated peptides, and IL-8 was measured by ELISA the next day. **G.** Biotinylated PRD and TAD peptide inhibit the LPS response in THP-1 cells. THP-1 cells were stimulated with LPS (1µg/ml) as indicated, and IL-8 was measured by ELISA the next day. **H.** Shorter PRD, TAD, and ZF7 peptides have the inhibitory activity. HCT-8 cells were stimulated overnight as indicated and IL-8 was measured by ELISA the next day. **I.** PRD, TAD, and ZF7 peptides inhibit the LPS response in RAW267.4 cells. RAW cells were stimulated with LPS (200ng/ml) without or with the indicated peptide (2g/ml) for 4h in the presence of monensin, and the TNF level was measured by flow cytometry.

A

B

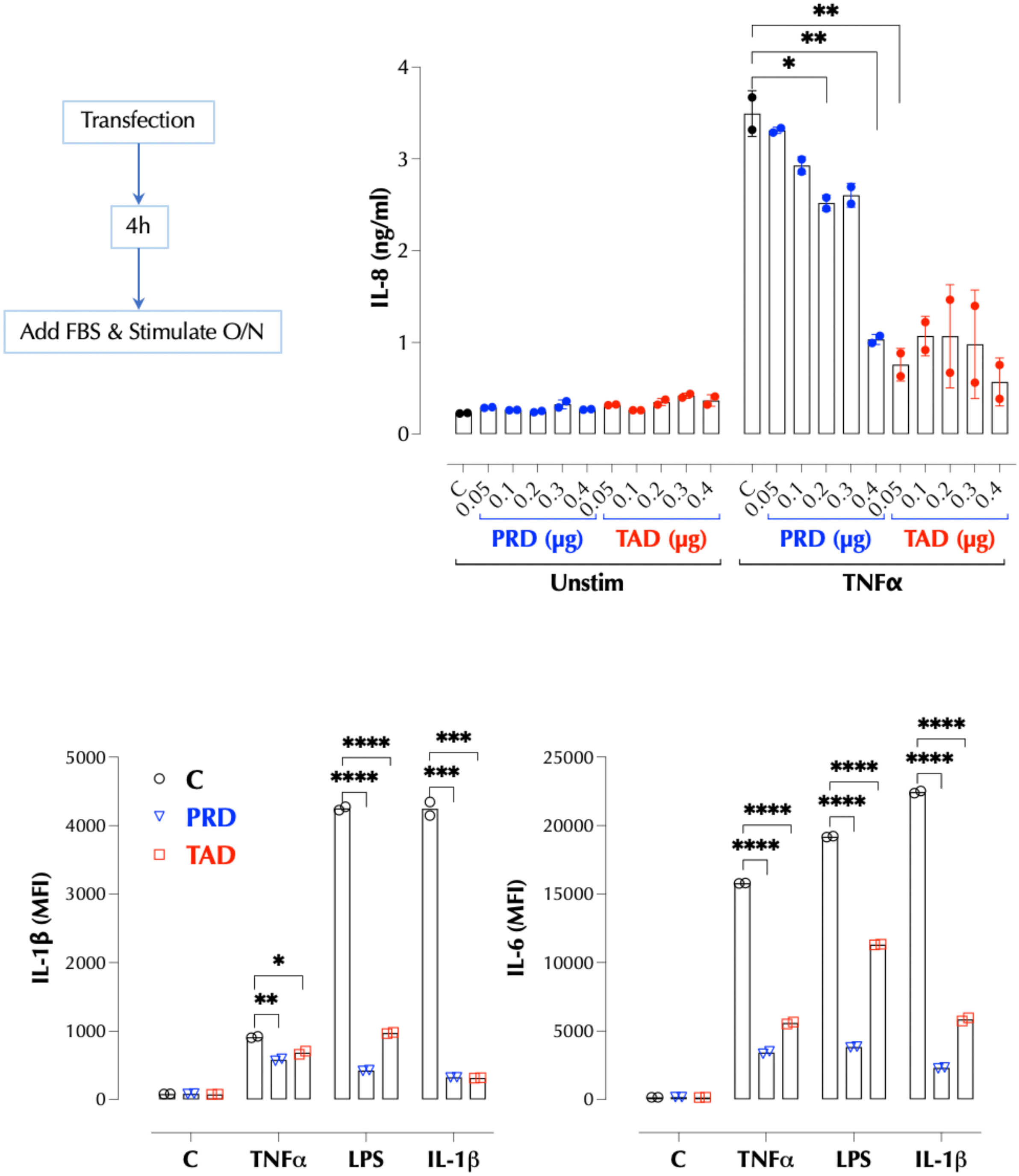
Transfection
4h
Add FBS & Stimulate O/N
IL-8 (ng/ml)
0
1
2
3
4
*
**
**
C
0.05
0.1
0.2
0.3
0.4
PRD (µg)
TAD (µg)
Unstim
TNFα
C
PRD
TAD
IL-1β (MFI)
0
1000
2000
3000
4000
5000
**
*
****
****
***
***
TNFα
LPS
IL-1β
IL-6 (MFI)
0
5000
10000
15000
20000
25000
****

C

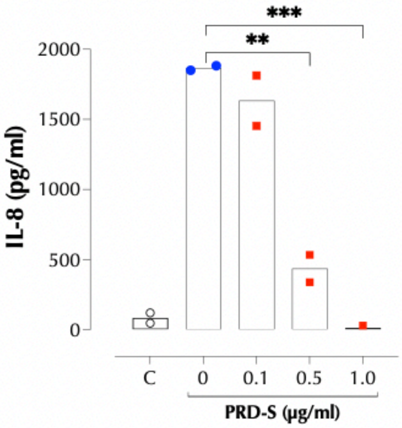

D

- **Control peptide:** ARNDCEQGHILKMFPSTWYV
- **Biotin-PRD**: **biotin**-**G**-GQPPSQPPQGQGPPSGPGQPAPAATQAAPQ
- **Biotin-TAD**: **biotin**-**G**-RQAMRININPSTANSPKCQE

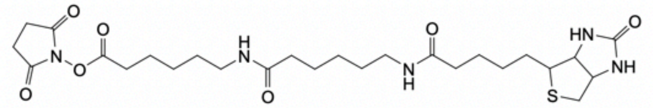

succinimidyl-6-(biotinamido)-6-hexanamido hexanoate

E

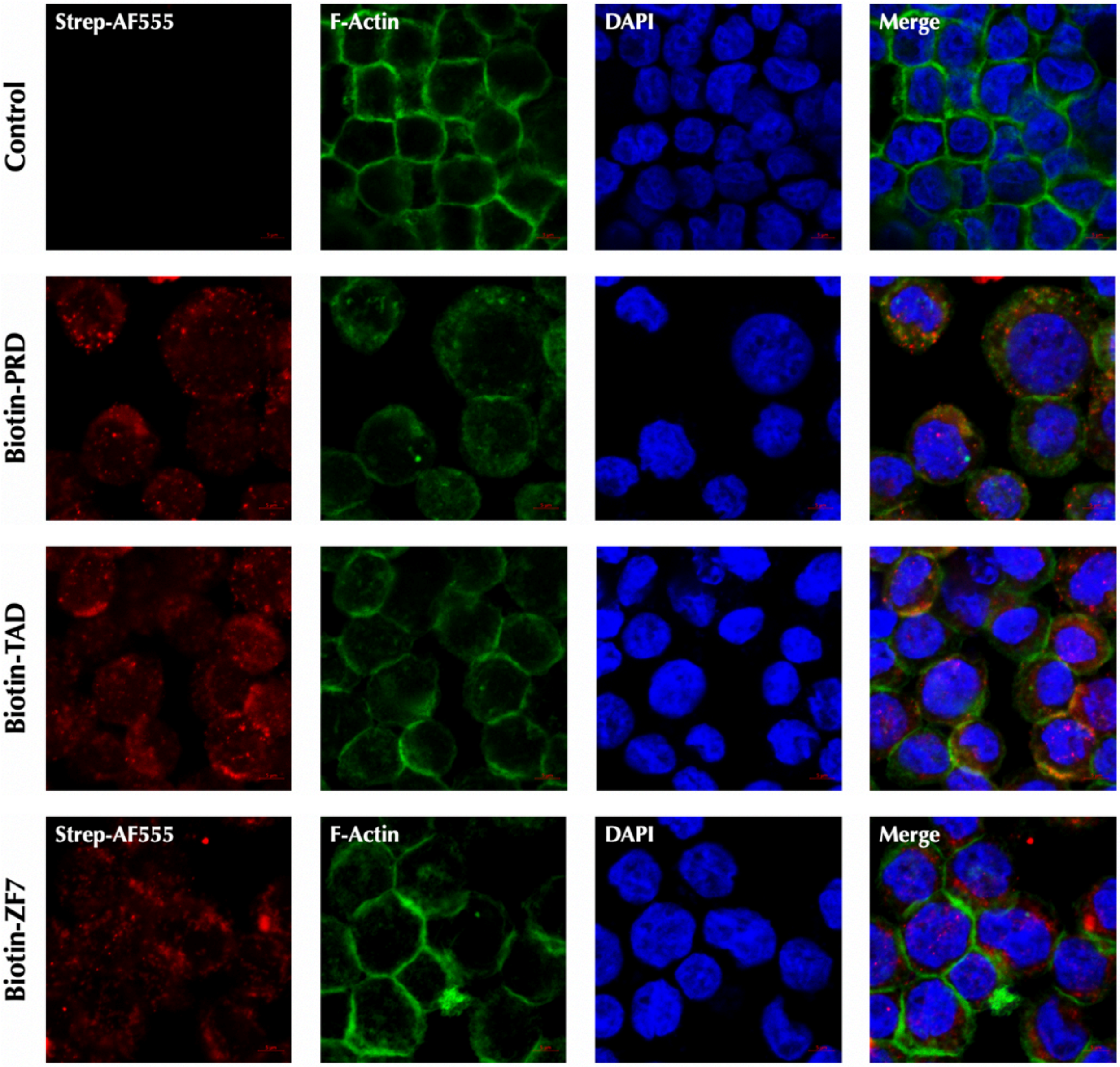
Strep-AF555
F-Actin
DAPI
Merge
Control
Biotin-PRD
Biotin-TAD
Strep-AF555
F-Actin
DAPI
Merge
Biotin-ZF7

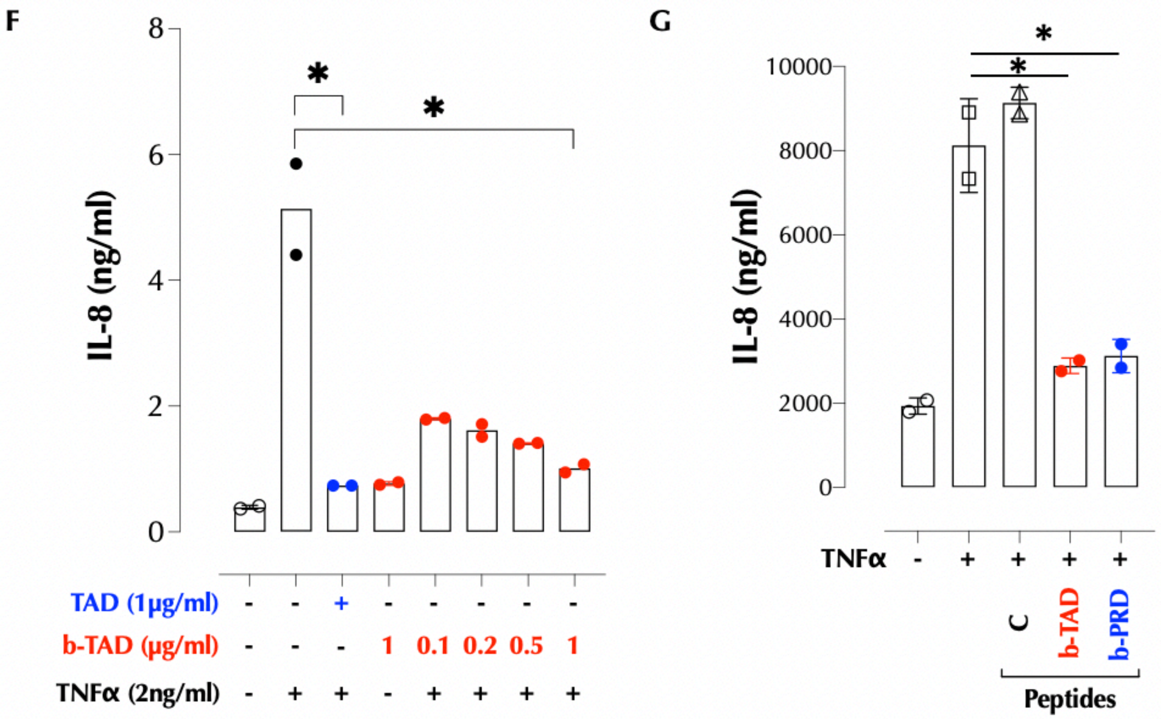

F
IL-8 (ng/ml)
0
2
4
6
8
*
*
TAD (1µg/ml) - - + - - - - -
b-TAD (µg/ml) - - - 1 0.1 0.2 0.5 1
TNFα (2ng/ml) - + + - + + + +
G
IL-8 (ng/ml)
0
2000
4000
6000
8000
10000
*
*
TNFα - + + + +
C
b-TAD
b-PRD
Peptides

H

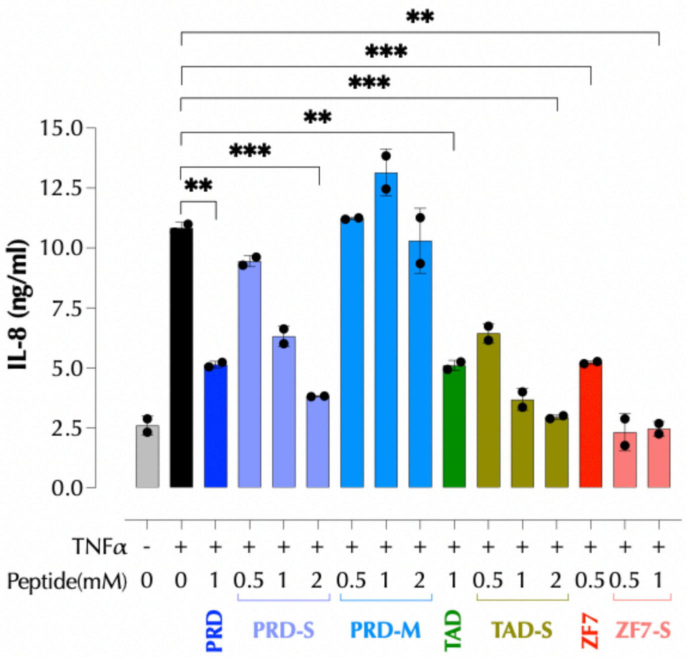

	Length (AA)	MW	Sequence	Activity
PRD	30	2802	GQPPSQPPQGQGPPSGPGQPAPAATQAAPQ	Yes
PRD-S	15	1371	QPPGQGPPSGPGQPA	Yes
PRD-M	13	1203	QPPGQGPPSGPGQ	No
TAD	20	2258	RQAMRININPSTANSPKCQE	Yes
TAD-S	17	1845	AMRININPSTANSPKCQ	Yes
ZF7	34	3929	PPKQRCRAPACDHFGNAKCNGYCNECYQFKQMYG	Yes
ZF7-S	24	2708	RCRAPACDHFGNAKCNGYCNECFQ	Yes

S: Short, M: Minimal

I

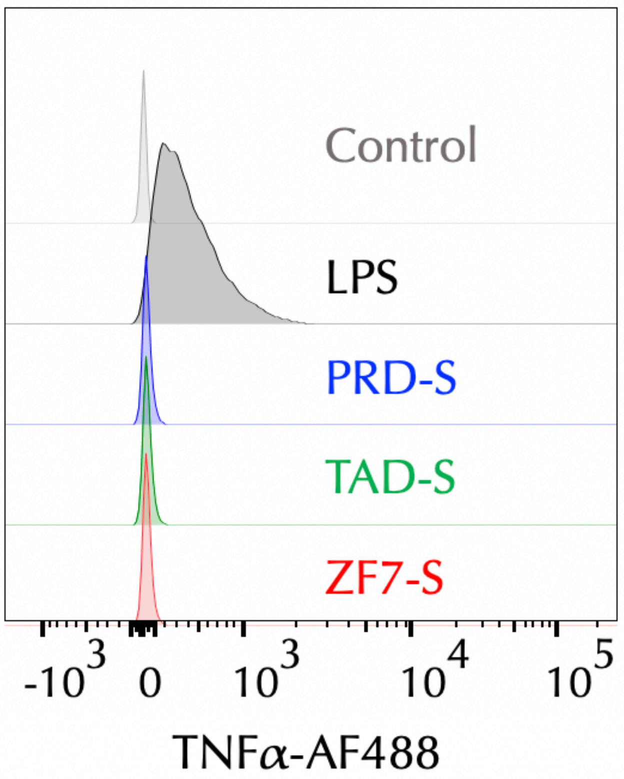

Supplemental Figure 5. TNFR1/RIP/TAK1 form a signaling complex.

293-T cells were transfected with TNFR1-HA, HA-RIP, and Myc-TAK1, and IP with either anti-TNFR1 or -HA antibody and WB were performed as indicated. Myc-tag in TAK1 was not detectable, suggesting a potential degradation or modification in the N-terminus.

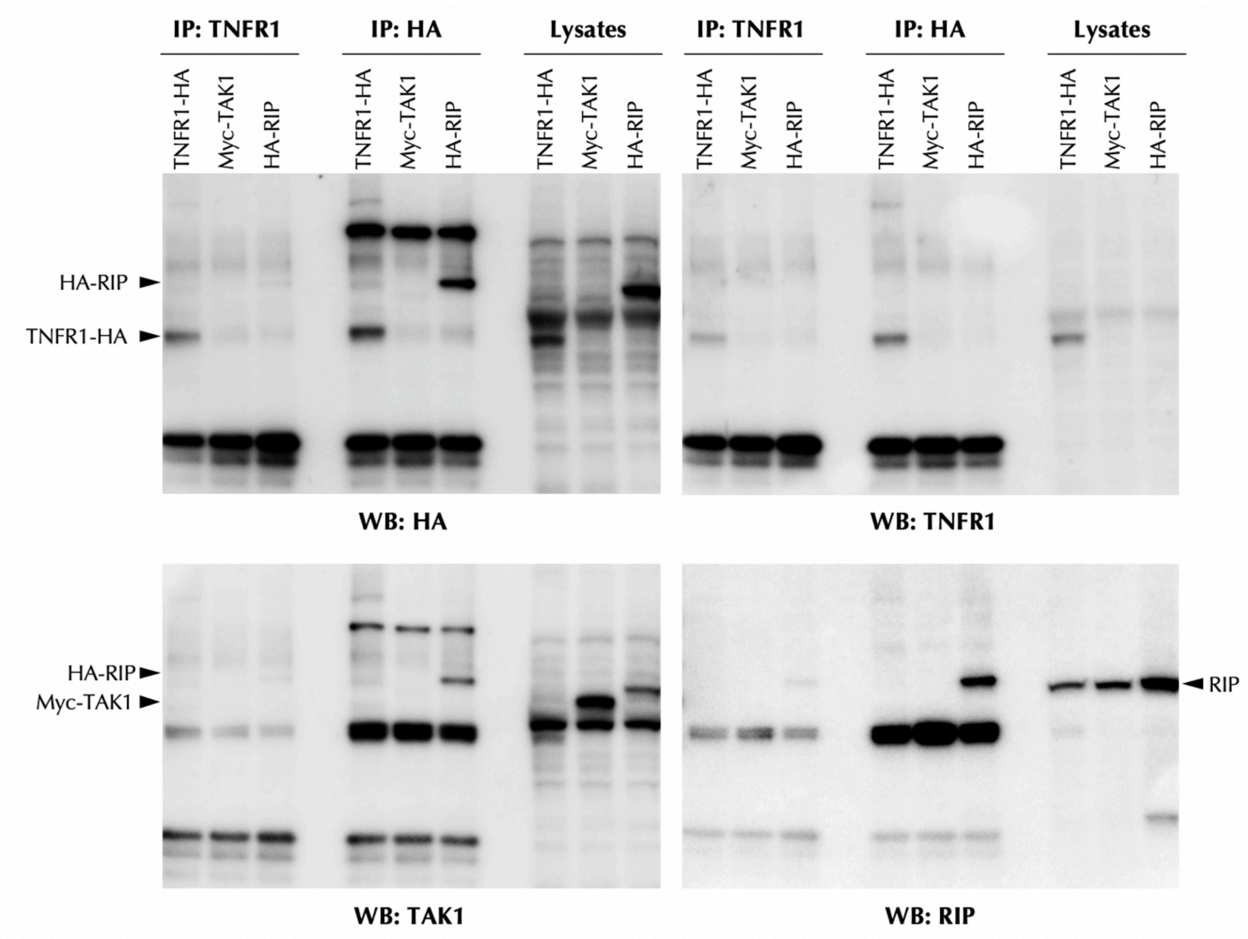

Supplement: Supplementary file 1 [file DataSheet_1.pdf]
